# The interplay between autophagy and immunogenic cell death: nanomaterial-based strategies for cancer immunotherapy

**DOI:** 10.1186/s12951-026-04286-5

**Published:** 2026-04-12

**Authors:** Mehdi Khorrami, Mahmood Fadaie, Saeid Razavi Dizaji, Akbar Davoodi, Rahim Asghari, Jianliang Shen, Ilnaz Rahimmanesh, Gautam Sethi, Pooyan Makvandi

**Affiliations:** 1 Department of Genetics and Molecular Biology, School of Medicine, Isfahan University of Medical Sciences, Isfahan, Iran; 2 Skin Diseases and Leishmaniasis Research Center, Isfahan University of Medical Sciences, Isfahan, Iran; 3 Department of Internal Medicine, Faculty of Medicine, Urmia University of Medical Sciences, Urmia, Iran; 4 Department of Mathematics and Computer Science, University of Southern Denmark, Odense, 5230 Denmark; 5 Hematology, Immune Cell Therapy, and Stem Cells Transplantation Research Center, Clinical Research Institute, Urmia University of Medical Sciences, Urmia, Iran; 6 Zhejiang Key Laboratory of Ophthalmic Drug Discovery and Medical Device Research, Eye Hospital, Wenzhou Medical University, Wenzhou, 325027 China; 7 Zhejiang Engineering Research Center for Tissue Repair Materials, Wenzhou Institute, University of Chinese Academy of Sciences, Wenzhou, 325001 China; 8 Applied Physiology Research Center, Cardiovascular Research Institute, Isfahan University of Medical Sciences, Isfahan, Iran; 9 Department of Pharmacology and NUS Centre for Cancer Research (N2CR), Yong Loo Lin School of Medicine, National University of Singapore, Singapore, 117600 Singapore; 10 Quzhou People’s Hospital, The Quzhou Affiliated Hospital of Wenzhou Medical University, Quzhou, 324000 Zhejiang China; 11 University College, Korea University, 02841 Seoul, Korea, Republic of (South Korea); 12 Centre for Research Impact and Outcome, Chitkara University, Rajpura, 140401 Punjab India

**Keywords:** Cancer immunotherapy, Immunogenic cell death, Autophagy, Nanotechnology

## Abstract

Cancer immunotherapy has significantly advanced the field of oncology. However, effectiveness of immunotherapy remains limited by tumor heterogeneity, immune evasion, and immunosuppressive tumor microenvironments. Immunogenic cell death (ICD) and autophagy are two interconnected biological mechanisms that play crucial roles in modulating tumor-immune interactions. ICD enhances antitumor immunity through the release of damage-associated molecular patterns (DAMPs), while autophagy affects the immunogenicity and viability of tumor cells in a context-dependent manner. Their complex interaction offers a distinctive platform to enhance immune responses and overcome resistance to immunotherapy. This review emphasizes recent advancements in using nanomaterials to regulate autophagy and ICD in cancer therapy. Various nanoplatforms, including metallic, polymeric, lipid-based, and carbon-based nanoparticles, have been engineered to deliver ICD inducers and autophagy modulators in a targeted TME-responsive manner. We discuss the underlying mechanisms, therapeutic synergies, and translational advantages of these dual-functioning systems. Furthermore, we address critical challenges such as biosafety, tumor specificity, and regulatory hurdles, and we explore strategies including PEGylation, biomimetic coating, and biodegradable carriers to improve clinical applications.

**Graphical Abstract:**

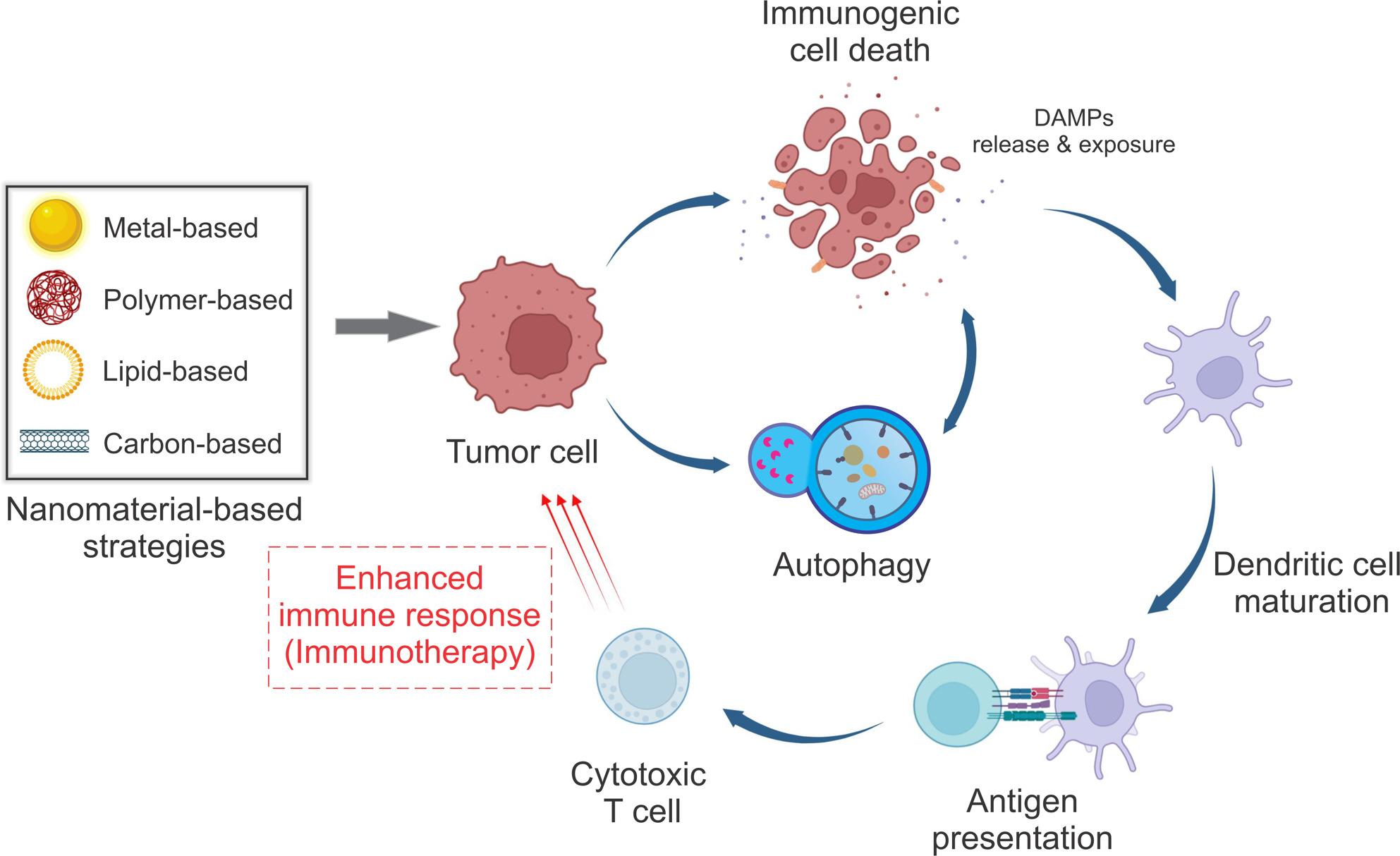

**Supplementary Information:**

The online version contains supplementary material available at 10.1186/s12951-026-04286-5.

## Introduction

Cancer remains a significant global health threat due to its heterogeneity, adaptability, and ability to evade conventional treatments [1]. Cancer immunotherapy, especially immune checkpoint inhibitors (ICIs) and chimeric antigen receptor T-cell therapy, has revolutionized oncology by using the immune system to identify and eradicate tumor cells [2, 3]. Nevertheless, the success of these therapies is still hampered by several factors: the ability of tumors to escape immune surveillance, the presence of immunosuppressive elements in the tumor microenvironment (TME), and the heterogeneity in how patients respond to treatment. These limitations highlight the urgent necessity for innovative strategies that enhance tumor immunogenicity and tackle resistance to immunotherapy.

Immunogenic cell death (ICD) represents a promising immunotherapeutic strategy by activating tumor cells to function as an intrinsic vaccine [4, 5]. In ICD, a regulated form of cell death, damage-associated molecular patterns (DAMPs) such as calreticulin (CRT), ATP, and High Mobility Group Box 1 (HMGB1) are released. These signals activate dendritic cells (DCs) and boost cytotoxic T lymphocyte responses [6]. ICD may be triggered by radiation, chemotherapy, and oncolytic viruses; however, these modalities often suffer from limited specificity and potential systemic toxicity [7]. Therefore, developing targeted and customized ICD inducers is essential to improve immunotherapy efficacy.

Autophagy, a highly regulated cellular degradation process, plays a dual and context-dependent role in cancer. Autophagy has the potential to prevent tumor formation by preserving cellular homeostasis. However, in pre-existing tumors, it frequently facilitates the survival of cancer cells in stressful conditions, thereby promoting therapeutic resistance [8]. Notably, autophagy modulates ICD by facilitating DAMP release, antigen processing, and immunological activation [9, 10]. The intricate interplay between autophagy and ICD suggests that the synchronized regulation of both processes may enhance the efficacy of immunotherapy, particularly in tumors that respond poorly to ICIs [11].

Nanotechnology offers a multifaceted platform to use this interplay. Nanomaterials, such as liposomes, polymeric nanoparticles, and inorganic platforms, can be designed to regulate autophagy and/or induce ICD in a precise, tumor-specific manner [12–14]. For example, stimuli-responsive nanoparticles can administer autophagy inducers like rapamycin or co-deliver chemotherapeutics that provoke ICD within the immunosuppressive TME, thereby boosting immune activation and synergizing with ICIs [15]. Numerous preclinical studies have illustrated the potential of such nanoplatforms in models of melanoma, triple-negative breast cancer (TNBC), and glioma. However, translational obstacles, such as biosafety and tumor-specific targeting, remain significant challenges [16].

This study explores the interplay between autophagy and ICD, with a focus on nanomaterials-based strategies designed to modulate both processes to enhance cancer immunotherapy. We examine the mechanistic basis of autophagy and ICD, their functional interactions within the TME, and recent nanotechnological advances that enable their dual modulation. Furthermore, we discuss current translational challenges and future directions, highlighting the potential of bioinspired nanomaterials, TME-responsive systems, and personalized approaches to shape the next generation of immunotherapies for treatment-resistant cancers.

## Immunogenic cell death (ICD) in cancer immunotherapy

ICD is a programmed cell death that elicits an immune response through the release of DAMPs within the TME [9, 17]. In contrast to conventional cell death, such as apoptosis, which typically does not elicit an immune response, ICD transforms dying tumor cells into a vaccine-like entity. This enhances anticancer immunity through the maturation of DCs and the activation of cytotoxic T cells [18].

ICD can trigger an adaptive immune response via a sequence of coordinated molecular processes [19]. The characteristics of ICD include the surface exposure of calreticulin (CRT) [20], the extracellular release of adenosine triphosphate (ATP) [21], high-mobility group box 1 (HMGB1) [22], and heat shock proteins (HSPs), including HSP70 and HSP90 [23]. These DAMPs function as “eat-me” or “find-me” signals, facilitating the phagocytosis by DCs, antigen presentation, and subsequent activation of cytotoxic T-cells [24]. For instance, CRT exposure on tumor cell surfaces enhances DC-mediated phagocytosis by binding to CD91 receptors on DCs, therefore activating NF-kB signaling and promoting the release of proinflammatory cytokines for Th17 priming [25]. ATP serves as a chemoattractant, attracting immune cells to the TME, where it interacts with P2Y2 and P2RX7 receptors to activate NLRP3 inflammasomes, resulting in the generation of IL-1β essential for IFN-γ-producing CD8^+^ T cells [26]. Upon release, HMGB1 binds to Toll-like receptor 4 (TLR4) on DCs, increasing antigen cross-presentation and T-cell activation [25]. Recent research has uncovered additional molecular mechanisms involved in ICD. Type I interferon (IFN) signaling is recognized as a vital element that enhances the immunogenicity of dying cells by facilitating chemokine synthesis and immune cell infiltration, with therapeutic significance shown in anthracycline-treated patients through TLR3 signaling [27–29]. The endoplasmic reticulum (ER) stress response, particularly the phosphorylation of eukaryotic initiation factor 2α (eIF2α), is a key upstream mechanism in CRT exposure, triggered by substantial ER stress resulting from the accumulation of misfolded proteins [30, 31]. For instance, researchers found that inhibiting the proteasome and IRE1-XBP1 axis of the ER stress response can improve myeloproliferative neoplasms phenotype caused by mutant CRT, suggesting more sustained treatment for eradicating disease-propagating myeloproliferative neoplasms [31]. These findings emphasize the complex nature of ICD and reinforce the necessity for accurate control to optimize its immunostimulatory effects.

**Fig. 1:**
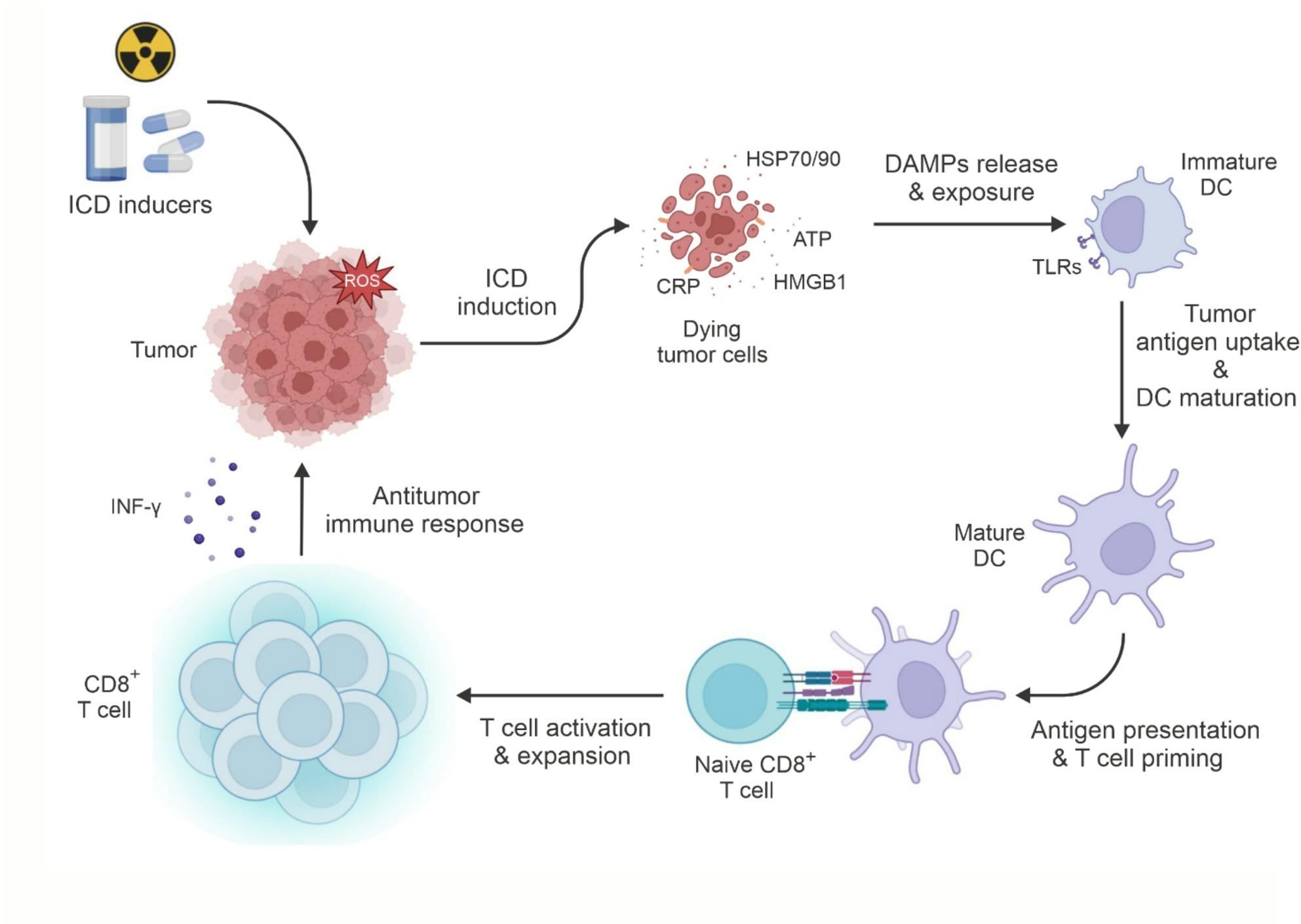
Schematic illustration of Immunogenic Cell Death (ICD) mechanism and subsequent T cell priming. ICD inducers (e.g., chemotherapy, radiation) trigger dying tumor cells to emit damage-associated molecular patterns (DAMPs) such as surface-exposed calreticulin (CRT) and released ATP and HMGB1. These signals act as “eat-me” and “find-me” flags, promoting the phagocytosis of tumor antigens by immature dendritic cells (DCs) and driving their maturation. Mature DCs then migrate to lymph nodes, where they present tumor antigens to naïve CD8 + T cells, priming them. The activated, antigen-specific CD8 + T cells subsequently infiltrate the tumor and execute an anti-tumor immune response, effectively turning the dying tumor cell into an in situ vaccine

ICD can be induced by many internal and external triggers such as chemotherapeutic drugs, radiation, photodynamic treatment (PDT), and oncolytic viruses [32, 33]. Anthracyclines (e.g., doxorubicin, mitoxantrone), oxaliplatin, and cyclophosphamide are well-established chemotherapeutic ICD inducers, increasing DAMP release and triggering anti-tumor immunity in preclinical models, with validation of their function in CRT exposure, ATP secretion, and HMGB1 release [34]. Retrospective studies also show that ICD inducers like anthracyclines, taxanes, and oxaliplatin improve clinical outcomes in breast, gastric, esophageal, and colorectal cancers, often correlating with immune infiltration, DC activation, and increased CRT exposure or eIF2α phosphorylation [35–37]. These markers are linked to better survival in cancers like acute myeloid leukemia, non-small cell lung cancer, and ovarian cancer. Cardiac glycosides, which induce ICD, also enhance survival when combined with non-ICD chemotherapies as shown in Fig. 1 [35].

Prospective trials further validate ICD inducers, including pegylated liposomal doxorubicin combined with atezolizumab for TNBC (ClinicalTrials.gov NCT03164993), doxorubicin with pembrolizumab [38], and lurbinectedin for small-cell lung cancer [39], which received FDA/EMA approval [40]. Other drugs such as belantamab mafodotin and PT-112 have potential; nevertheless, challenges like toxicity and the absence of ICI combination trials remain [41]. LTX-315 is now undergoing clinical investigation, underscoring continuous endeavors to enhance ICD-based therapeutics [42, 43].

ICD is induced by radiation via the generation of reactive oxygen species (ROS) and ER stress, whereas PDT employs light-activated photosensitizers to trigger localized tumor cell death and release DAMP [44, 45]. Oncolytic viruses, such as talimogene laherparepvec (T-VEC), induce ICD by directly lysing tumor cells and triggering antiviral immune responses that cross-prime anti-tumor immunity [46]. Research on T-VEC revealed that it induces ICD and facilitates the maturation of human BDCA-1^+^ myeloid DCs (Blood Dendritic Cell Antigen 1), highlighting its potential in cancer immunotherapy [47]. Another study revealed that the sensitivity of melanoma cells to T-VEC was negatively correlated with stimulator of interferon genes (STING) expression. The CRISPR/Cas9-STING knockdown correlated with enhanced T-VEC cell killing [48]. T-VEC elicits ICD in vitro, enhances tumor immunity, and can cause therapeutic responses in anti-PD-1-refractory melanoma with low STING expression. Notably, it was shown that high hydrostatic pressure (HHP) induces ICD in cancer cells, presenting a potential physical strategy for ex vivo vaccine production [49]. A study demonstrated that HHP trigger a specific antitumor immune response, causing the rapid translocation of CRT to the plasma membrane surface (ecto-CRT) [50]. One important biological factor that differentiates immunogenic from non-ICD is ecto-CRT. The research concluded that the idea of HHP-driven cell death as immunogenic is based on the ROS– PERK )Protein kinase RNA-like endoplasmic reticulum kinase(-eIF2α (eukaryotic initiation factor-2α)-caspase 2-caspase 8-caspase 8-mediated cleavage of the ER protein BAP31 (B-cell receptor-associated protein 31) [50]. The study concluded that PERK plays a central role in the exposure of CRT upon ICD induction. Additionally, small molecule inhibitors targeting particular pathways, such as the cGAS-STING pathway, have been found to be effective ICD inducers via boosting type I IFN signaling [51]. The cGAS-STING pathway, indeed, boosts innate and adaptive immunity through the activation of DCs, T cells, natural killer cells, and macrophages, with STING serving as a vital transducer. Immune cell infiltration and STING expression in tumors positively correlate in the Cancer Genome Atlas Program (TCGA) database [52]. These studies show the rising range of ICD-inducing techniques, although their clinical applicability remains confined by many limitations.

Traditional ICD inducers face substantial challenges, despite their potential. The lack of tumor selectivity in chemotherapeutic drugs, such as anthracyclines, causes systemic toxicity and off-target effects on healthy tissues [53, 54]. Radiotherapy and PDT are hampered by their dependence on tumor accessibility and the difficulty of treating metastatic tumors adequately [55, 56]. Although oncolytic viruses are promising, they encounter challenges in the areas of viral delivery, immunological clearance, and industrial scalability [57]. Moreover, the efficiency of ICD inducers is typically impaired by the immunosuppressive TME, which can reduce DAMP-mediated immune activation through mechanisms such as TGF-β signaling or myeloid-derived suppressor cell activity [58, 59]. Recent studies have also demonstrated diversity in ICD development among tumor types and patient cohorts. For example, studies indicate that some cancers with poor antigenicity or deficient DAMP signaling pathways are resistant to ICD-mediated immune activation [60, 61], reflecting defective ATP release owing to epigenetic alterations or CD39/CD73 overexpression [62]. This finding highlights the need for tailored, tumor-specific ICD inducers that can overcome TME immunosuppression and boost synergy with current immunotherapies.

ICD presents significant promise as a therapeutic target within the realm of cancer immunotherapy, especially when utilized in combination with immune checkpoint inhibitors (ICIs), including anti-PD-1/PD-L1 or anti-CTLA-4 antibodies [63, 64]. By enhancing tumor immunogenicity, ICD might sensitize “cold” tumors, those with little immune infiltration, to ICI treatment, expanding the pool of responsive patients. ICD-based strategies are also being investigated in cancer vaccines; HSP-enriched lysates are candidate vaccines that use ICD-induced tumor antigens to trigger immune responses [65]. Emerging clinical studies are testing ICD-focused treatments. A 2024 phase I study, for example, examined the combination of PDT with anti-PD-1 treatment in head and neck squamous cell carcinoma and found enhanced immune infiltration and partial responses in 60% of patients (ClinicalTrials.gov NCT04305795). Likewise, nanoparticle-based ICD inducers—such as those delivering chemotherapeutics or ROS-generating chemicals—are entering early-phase trials using nanotechnology’s accuracy to improve ICD effectiveness, therefore increasing tumor cell cytotoxicity [66–68]. These developments gain attention for the translational promise of ICD but also highlight the necessity of innovative strategies, including nanotechnology, to overcome the constraints of present inducers.

## Autophagy and Its crosstalk with ICD

Autophagy, a carefully controlled cellular mechanism for the degradation and recycling of cytoplasmic components, is a crucial factor influencing cancer cell fate and treatment efficacy. The intricate interplay of autophagy with ICD has become a critical focus in cancer immunotherapy [69, 70]. This section provides a thorough overview of the molecular principles of autophagy, its dysregulation in cancer, and its synergistic function in ICD.

### Molecular basis of autophagy in cancer

Autophagy, primarily macroautophagy, is a multifaceted process that preserves cellular homeostasis by digesting damaged organelles, misfolded proteins, and other cytoplasmic components [71]. The process initiates with the development of a phagophore, which extends to create a double-membrane autophagosome that encapsulates cellular material. Autophagosomes merge with lysosomes to create autolysosomes, wherein hydrolytic enzymes decompose contents, delivering amino acids, lipids, and carbohydrates to facilitate cellular metabolism [72]. Autophagy is carefully regulated by a network of autophagy-related genes (ATGs) and signaling pathways that respond to cellular stress. The molecular mechanism of autophagy is coordinated by more than 30 ATGs. Beclin-1 (ATG6), in conjunction with class III phosphatidylinositol 3-kinase (PI3K, also referred to as VPS34), commences phagophore nucleation by producing phosphatidylinositol 3-phosphate (PI3P), which attracts effector proteins such as WIPI2 (WD repeat domain, phosphoinositide-interacting protein 2) [73, 74]. ATG5, ATG12, and ATG16L1 form a conjugate complex that facilitates autophagosome membrane elongation, while LC3 (microtubule-associated protein 1 light chain 3) is lipidated with phosphatidylethanolamine (PE) by ATG3 and ATG7 to mark autophagosomal membranes. Soluble N-ethylmaleimide–sensitive factor attachment protein receptors (SNARE), e.g., Syntaxin 17 (STX17), SNAP29 (Synaptosomal-Associated Protein 29 kDa), and VAMP8 (Vesicle-Associated Membrane Protein 8), and the HOPS complex (Homotypic Fusion and Protein Sorting complex) drive autophagosome-lysosome fusion, hence ensuring effective cargo degradation. Nutrient and stress-sensing mechanisms control autophagy [75, 76]. Under nutrient-rich circumstances, the mTORC1 (mechanistic target of rapamycin complex 1) pathway suppresses autophagy by phosphorylating ULK1 (Unc-51-like kinase 1), a major activator of phagophore production. AMPK (AMP-activated protein kinase) stimulates ULK1 during nutrient deprivation or stress, therefore increasing autophagy by phosphorylating Beclin-1 and VPS34 [77, 78].

ER stress, hypoxia, or oxidative stress further stimulate autophagy through the unfolded protein response (UPR), especially via PERK-mediated phosphorylation of eIF2α, which enhances the expression of ATG genes [79]. Epigenetic regulation is significant, as histone acetyltransferases (e.g., p300) and deacetylases (e.g., SIRT1) modulate ATG expression in response to cellular stress [80]. The dual function of autophagy in cancer is governed by its molecular regulation. During the first stages of cancer, autophagy plays a crucial role in preventing malignant transformation by removing damaged mitochondria (mitophagy) and reducing the levels of ROS, thereby averting DNA damage [81]. Monoallelic loss of Beclin-1 or mutations in ATG5 in malignancies such as breast and ovarian carcinomas disrupt this protective mechanism, facilitating oncogenesis. Conversely, advanced cancers utilize autophagy to endure microenvironmental stressful conditions, including hypoxia and nutritional scarcity [82]. KRAS (Kirsten rat sarcoma viral oncogene homolog)-mutant lung tumors elevate autophagy through MAPK signaling, hence augmenting glutamine metabolism to support tumor proliferation [83]. Autophagy sustains cancer stem cells (CSCs) by upregulating SOX2 (sex determining region Y-Box Transcription Factor 2) and NANOG, hence facilitating tumor recurrence [84].

The molecular interaction of autophagy with ICD is essential. Autophagy promotes ATP secretion, an essential damage-associated molecular pattern in ICD, by delivering ATP to the plasma membrane through LC3-positive autophagosomes [85, 86]. Dysregulated autophagy can inhibit apoptosis, hence facilitating multidrug resistance (MDR) by eliminating pro-apoptotic signals, including the release of cytochrome c [87]. Recent investigations demonstrate the involvement of lncRNAs in the modulation of autophagy by miRNA sponging, connecting epigenetic regulation to therapeutic resistance and ICD efficacy [88, 89]. Comprehending these molecular pathways is crucial for targeting autophagy in cancer immunotherapy.

### Dysregulation of autophagy in cancer

A hallmark of cancer is autophagy dysregulation, which has a profound impact on tumor initiation, growth, metastasis, and treatment resistance. This dysregulation results from genetic, epigenetic, and microenvironmental changes that impair the homeostatic balance of autophagy, altering its function from tumor suppression to tumor promotion and immune evasion [90, 91]. These alterations have substantial ramifications for ICD, since they regulate the release of DAMPs essential for antitumor immunity. Mutations in ATGs are prevalent in malignancies [92, 93]. Monoallelic deletions of Beclin-1, identified in breast, ovarian, and prostate cancers, compromise autophagy’s tumor-suppressive role, resulting in the accumulation of damaged mitochondria and ROS, which foster genomic instability [94, 95]. Likewise, mutations in ATG5 or ATG7, reported in colorectal and gastric cancers, hinder autophagosome formation, thereby accelerating tumorigenesis by diminishing the clearance of oncogenic protein aggregates [96]. A study identified two somatic missense variants, in the *ATG5* gene encoding regions p.Leu112Phe and p.His41Tyr in gastric and hepatocellular carcinomas [97]. ATG5 protein was expressed in normal cells but lost in gastric, colorectal, and hepatocellular carcinomas. The data suggest that somatic mutations and loss of expression of the ATG5 gene might play a role in gastrointestinal cancer pathogenesis [97].

Oncogenic mutations such as KRAS or BRAF can activate the ERK/MAPK pathway, which in turn enhances autophagy and helps cancer cells survive nutrient limitation and chemotherapy stress [98, 99]. KRAS-driven pancreatic ductal adenocarcinomas display increased LC3-II levels, facilitating tumor metabolism via augmented mitophagy and amino acid recycling [100, 101]. Epigenetic alterations further intensify autophagy dysfunction. Hypermethylation of the Beclin-1 or ATG16L1 promoters, seen in hepatocellular carcinoma and leukemia, inhibits autophagy gene expression, hence facilitating tumor proliferation [102]. In contrast, the suppression of histone deacetylase (HDAC) by SIRT1 or lncRNA-mediated miRNA sponging (such as the HOTAIR-miR-34a axis) enhances autophagy in breast and lung malignancies, hence providing resistance to apoptosis-inducing drugs like doxorubicin [103, 104]. Research found the lncRNA MALAT1 (Metastasis-Associated Lung Adenocarcinoma Transcript 1) as a crucial regulator of autophagy in glioblastoma, promoting autophagic flux through the inhibition of miR-101, hence facilitating tumor cell survival under temozolomide therapy [105].

Microenvironmental factors, specifically hypoxia and lack of nutrients, induce autophagy dysregulation within TME. Hypoxia-mediated activation of HIF-1α stimulates BNIP3 (BCL2/adenovirus E1B 19-kDa-interacting protein 3) and BNIP3L(BCL2/adenovirus E1B 19 kDa-interacting protein 3-like), thereby promoting selective autophagy (such as mitophagy) to preserve redox homeostasis and aid metastasis [106, 107]. In metastatic melanoma, HIF-1α-driven autophagy enhances invadopodia formation, thereby facilitating the degradation of the extracellular matrix [108, 109].

TGF-β and Interleukin-10 (IL-10) in the TME enhance autophagy in cancer-associated fibroblasts and myeloid-derived suppressor cells (MDSCs), creating an immunosuppressive environment that diminishes the efficacy of ICD by augmenting regulatory T-cell (Treg) activity [110, 111]. Dysregulation of autophagy significantly influences therapy resistance and ICD. In MDR cancers, including TNBC, heightened autophagic flux plays a crucial role in the removal of damaged organelles and pro-apoptotic signals, thereby impeding caspase activation and the process of apoptosis [112]. For instance, the increased expression of LC3 and ATG7 in cisplatin-resistant ovarian cancer correlates with a reduction in cytochrome c release, thereby hindering chemotherapy-induced cell death [113].

Dysregulated autophagy can hinder DAMP release in the context of ICD. Epigenetic silencing of ATG5 in lymphoma decreases ATP production, hence impairing DC recruitment and immunological activation [114]. In contrast, increased autophagy in cancer stem cells, mediated by ALDH1A1 (Aldehyde Dehydrogenase 1A1) or SOX2, diminishes CRT exposure, thereby decreasing the immunogenicity of ICD and facilitating tumor recurrence [115, 116]. An investigation has shown that ITGB4 can bind to BNIP3 and promote the phagocytosis of MHC-I (Major Histocompatibility Complex class I) by autophagosomes. Downregulation of ITGB4 significantly improved the therapeutic effect of PD-1 antibodies on pancreatic cancer [117]. Autophagy is positively correlated with the ITGB4-BNIP3 complex protein expression level, diminishing MHC-I protein expression and promoting immune escape [117].

Tumor heterogeneity complicates the dysregulation of autophagy. Distinct tumor subclones have diverse autophagic dependencies, with cancer stem cells significantly dependent on autophagy for survival, whereas differentiated tumor cells may show less autophagy due to hyperactivation of mTORC1 [118, 119]. This heterogeneity complicates therapeutic targeting, since autophagy suppression may sensitize certain tumor cells to chemotherapy while diminishing ICD in others by decreasing ATP release [9, 120]. Recent advancements indicate that single-cell RNA sequencing can identify autophagic heterogeneity, revealing LC3-II and p62 expression as biomarkers for personalized therapy [121, 122].

Given the dual role of autophagy, the decision to inhibit or activate this process should be context-dependent. Autophagy activation is desirable when it promotes pro-death mechanisms and enhances ICD, leading to DAMP release and improved antitumor immunity [123, 124]. Conversely, autophagy inhibition is advantageous in tumors or subpopulations, such as cancer stem cells or cytoprotective tumor subclones, where autophagy supports survival and therapy resistance. Context-specific strategies, including spatially or temporally controlled autophagy modulation using nanomaterials, may allow selective pro-death autophagy in tumor cells while suppressing cytoprotective autophagy in resistant or immunosuppressive populations, maximizing ICD and therapeutic efficacy.

**Fig. 2:**
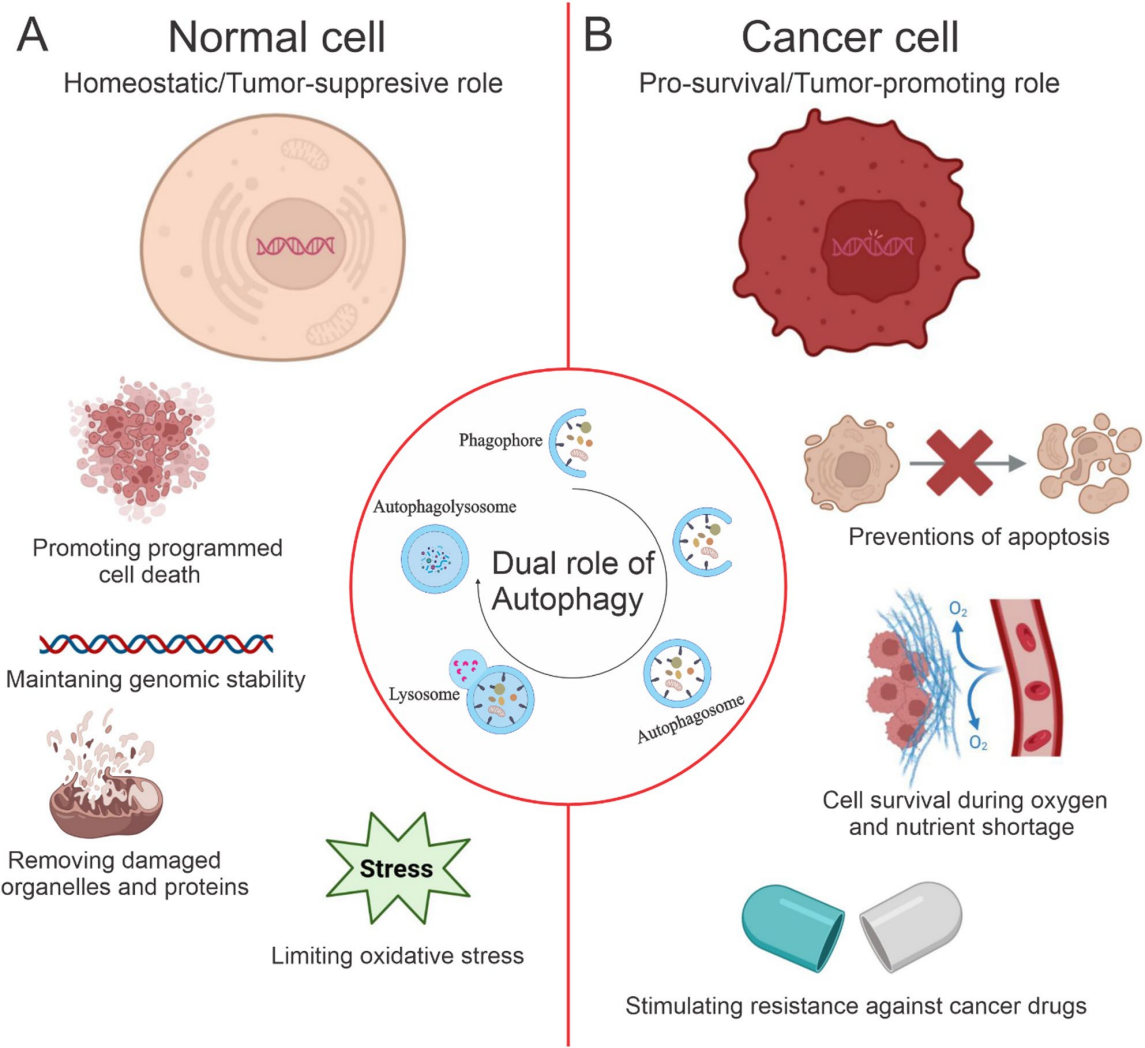
Schematic illustration of the dual role of autophagy in cancer. Under normal conditions (**A**), autophagy acts as a tumor-suppressive mechanism by maintaining genomic stability, removing damaged components, and promoting cell death. In cancer cells (**B**), dysregulated autophagy adopts a pro-survival, tumor-promoting role, enabling resistance to stress, preventing apoptosis, and fostering drug resistance

In summary, dysregulation of autophagy in cancer, impacted by factors such as genetics, epigenetics, and the microenvironment, turns autophagy into a process that promotes tumor growth rather than tumor suppression. This leads to an increase in immune evasion, metastasis, and multidrug resistance. The influence on ICD highlights the necessity for accurate regulation to reinstate DAMP release and enhance antitumor immunity, paving the way for personalized therapy approaches. Figure 2 shows a schematic illustration of the dual role of autophagy in cancer.

### Autophagy as a modulator of ICD-associated immunogenicity

Recent evidence suggests that autophagy can be essential for specific immunogenic features of ICD, particularly ATP secretion which serves as a crucial “find-me” signal that triggers immune responses [21, 125]. Extracellular ATP interacts with P2Y2 and P2RX7 receptors on DCs and macrophages, leading to the activation of the NLRP3 inflammasome and the release of IL-1β, which subsequently primes cytotoxic T lymphocytes (CTLs) for antitumor immunity [126–128]. Studies utilizing autophagy-deficient tumor models (e.g., Atg5 or Atg7 knockdown) indicate a marked decrease in ATP release, which hinders DC recruitment and T-cell activation, even with intact CRT exposure and HMGB1 release [129–131]. This finding highlights the specific function of autophagy in ATP-dependent immune activation.

The molecular process includes autophagy-dependent lysosomal exocytosis. During ICD, ATP accumulates within autophagosomes identified by LC3 (microtubule-associated protein 1 light chain 3) and co-localizes with lysosomal markers such as LAMP1 [132–135]. These autophagosomes mature into amphisomes, which subsequently fuse with the plasma membrane through the VAMP7 (vesicle-associated membrane protein 7)-SNARE complex, resulting in the extracellular release of ATP. ER stress, triggered by ICD inducers such as anthracyclines, enhances this process via phosphorylation of Beclin-1 by death-associated protein kinase (DAPK), thereby promoting autophagosome biogenesis [136]. Studies indicate that lysosomal acidification, regulated by mTORC1 inhibition and TFEB activation, is essential for efficient ATP exocytosis, thereby connecting metabolic signaling to ICD [137–139].

In addition to its established degradative function, autophagy actively modulates extracellular ATP release during ICD via lysosomal trafficking and calcium-dependent lysosomal exocytosis. Nanomaterials can affect this process by altering lysosomal pH, membrane integrity, intracellular Ca²⁺ flux, and cytoskeletal dynamics, all of which regulate lysosomal mobility and fusion efficacy with the plasma membrane [9, 85]. Nanoparticle-induced lysosomal stress or transitory lysosomal permeabilization may promote ATP secretion and augment immune cell recruitment, whereas severe lysosomal dysfunction or obstruction of autophagic flux can inhibit ATP-mediated immunogenic signaling [140, 141]. This lysosome-centered regulatory axis, crucial for connecting autophagy to ICD, is inadequately examined in the context of nanomaterial-based therapeutic design and signifies a vital avenue for future research.

Autophagy also affects other DAMPs, but to a smaller degree. For instance, autophagy facilitates HMGB1 release by promoting lysosomal integrity during cell death, ensuring its extracellular availability to bind TLR4 on DCs [142, 143]. CRT exposure, driven by ER stress and eIF2α phosphorylation, is primarily autophagy-independent; however, autophagy may improve its surface presentation by preserving ER homeostasis [144, 145]. Recent findings indicate that autophagy modulates type I interferon (IFN) signaling, an essential ICD component, by eliminating damaged mitochondria. This process relieves the suppression of the cGAS-STING pathway and enhances chemokine production [146]. A study demonstrated that the proteasome inhibitor bortezomib (BTZ), in addition to its known cytotoxic effects, induces ICD in multiple myeloma cells by enhancing CRT exposure and facilitating phagocytosis by DCs, which in turn activates tumor-specific T cell responses [147]. This immune-stimulatory effect is mediated through the cGAS/STING pathway, which senses BTZ-induced cytosolic DNA and initiates an IFN response, enhancing T-cell infiltration and anti-tumor immunity. The study identifies an ICD gene signature associated with improved clinical outcomes in BTZ-treated patients and shows that combining BTZ with STING agonists like ADU-S100 potentiates immune activation and tumor regression [147].

Enhancing autophagy-dependent ATP release presents therapeutic potential. Nucleotidase inhibitors such as ARL67156 inhibit ATP degradation by CD39/CD73, thereby maintaining immune activation [148]. Furthermore, the combination of autophagy inducers with ICD agents such as lurbinectedin promotes the release of ATP and HMGB1, as shown in current clinical trials in small-cell lung cancer [149].

**Fig. 3:**
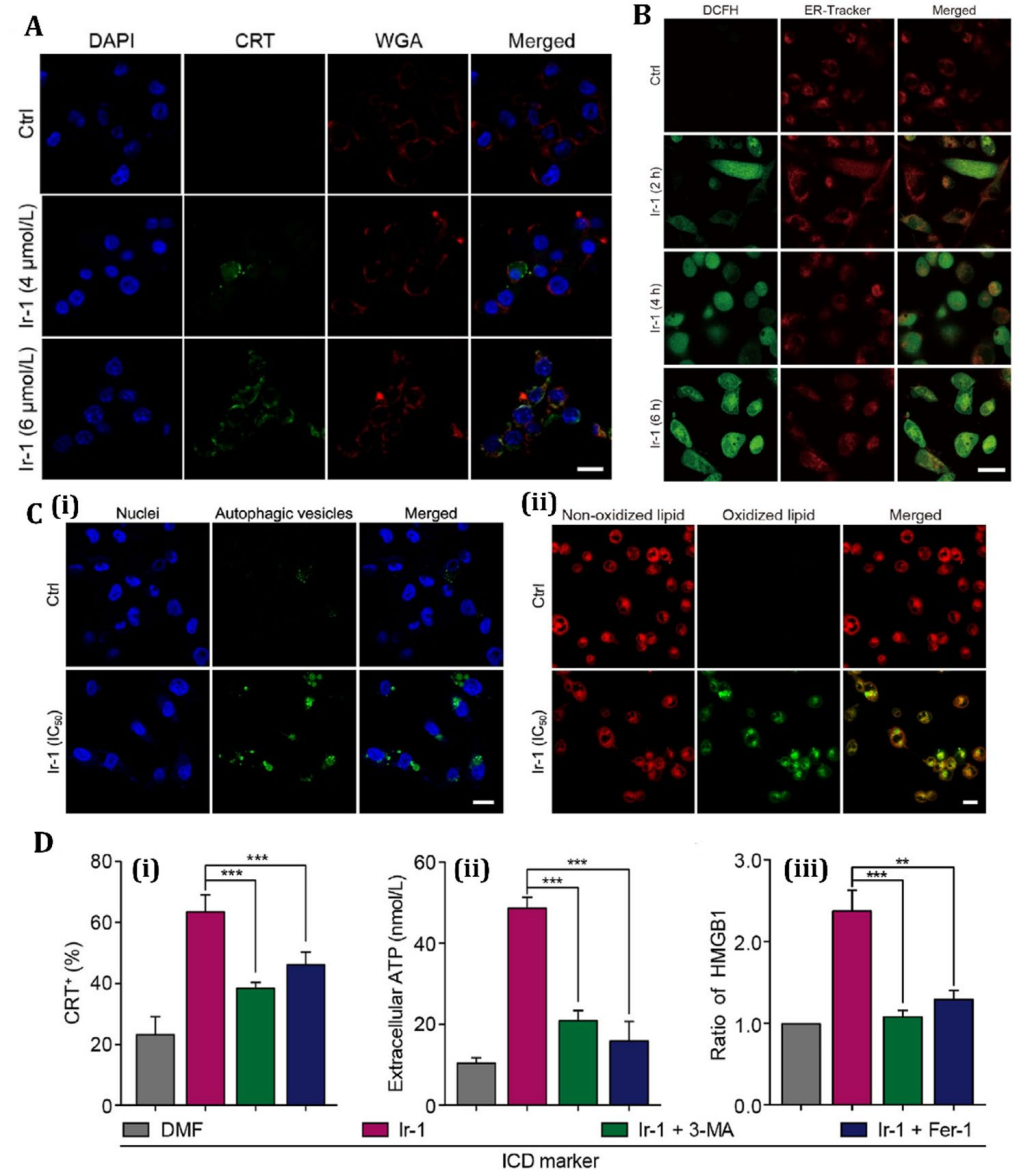
Multifaceted effects of Ir-1 on MDA-MB-231 cells by inducing autophagy and ICD. **(A)** Confocal imaging illustrates calreticulin (CRT) exposure following 12-hour treatment with Ir-1, indicative of immunogenic cell death (ICD). **(B)** Visualization of intracellular ROS generation and ER stress induced by Ir-1 at its IC50 concentration. **(C)** Ir-1-induced autophagy and ferroptosis: **(i)** Formation of autophagic vesicles (green) with DAPI-stained nuclei (blue); scale bar = 20 μm. **(ii)** Lipid peroxidation indicating ferroptotic activity. **(D)** Role of ROS in modulating autophagy, ferroptosis, ICD, and IDO suppression: **(i)** Surface CRT expression quantified via flow cytometry after co-treatment with Ir-1 and either 3-MA or Fer-1. **(ii**,** iii)** Levels of extracellular ATP **(ii)** and HMGB1 **(iii)** following 12-hour co-treatment, reflecting ICD-associated DAMP release. Reproduced from [150] with permission from Elsevier

Recent advances have identified novel autophagy-dependent ICD inducers with potential for overcoming challenges in cancers like TNBC, where traditional ICD inducers show limited efficacy. An iridium (III) complex, Ir-1, has been demonstrated to induce autophagy-dependent ferroptosis in triple-negative breast cancer cells (MDA-MB-231), resulting in a ferroptosis-mediated ICD response marked by ROS-induced ER stress and the secretion of DAMPs, including ATP and HMGB1 (Fig. 3) [150]. This process stimulates CD8^+^ T-cell responses and reduces Foxp3+ regulatory T cells, providing enduring anticancer immunity in immunocompetent BALB/c mice. Ir-1 significantly inhibits indoleamine 2,3-dioxygenase, hence augmenting the synergy with anti-PD-1 treatment to enhance therapeutic efficacy in TNBC models [150]. Similarly, another iridium (III) complex, 2a, modified with an oxoisoaporphine alkaloid, causes autophagy-dependent ICD by targeting the lysosomal protease cathepsin D (Cat D) [151]. Thermal proteome profiling and biochemical experiments revealed that 2a inhibits Cat D, hence activating the LKB1 (Liver Kinase B1)-AMPK(AMP-activated protein kinase)-ULK1 (Unc-51-like autophagy activating kinase 1) signaling pathway to promote autophagy and ICD. This method, unlike previous ICD inducers, identifies Cat D as a new molecular target for autophagy-mediated ICD. Conversely, a similar analogue, 1a, promotes ICD through ER stress by destabilizing binding immunoglobulin protein (BiP), demonstrating the variability of autophagy-ICD pathways [151]. These findings highlight the potential of iridium-based complexes as precise ICD inducers, broadening treatment approaches for immunotherapy-resistant malignancies such as TNBC.

Notably, many studies report concurrent increases in autophagy markers (e.g., LC3-II accumulation and p62 degradation) and ICD hallmarks, including surface calreticulin exposure, extracellular ATP, and HMGB1 release, following treatment with diverse stressors such as nanomaterial-delivered agents. However, correlation alone does not establish causation, and a direct autophagy-ICD link requires evidence that selective autophagy inhibition reduces these immunogenic features. Indeed, genetic ablation of core autophagy genes (e.g., ATG5 or ATG7) or inhibition of early autophagy steps impairs ATP secretion and weakens therapy-induced antitumor immunity, supporting a permissive role for autophagy in ATP-dependent ICD pathways [141]. In contrast, blockade of late-stage autophagy (e.g., chloroquine or bafilomycin A1) may enhance ICD under certain conditions by preventing lysosomal degradation of DAMPs or antigen-presentation machinery. Together, these findings highlight the context- and stage-dependent nature of autophagy-ICD crosstalk, indicating the need for precise, stimuli-responsive nanomaterial strategies to selectively harness pro-immunogenic effects while avoiding unintended immunosuppression.

In practice, the induction of autophagy is typically preferred in immunologically “cold” tumors or under mild stressors, such as specific chemotherapeutics or sonodynamic therapy in hypoxic areas, where early autophagy promotes ATP and CRT release, along with antigen processing, without providing a significant survival advantage. Conversely, inhibiting late-stage autophagy is more beneficial in advanced, resistant tumors, cancer stem cell-dense niches, or highly autophagic microenvironments, where protective autophagy might diminish immunogenic signals and foster therapeutic resistance. TME-responsive nanoplatforms, activated by pH, redox alterations, or hypoxia, provide a systematic method for stage- and context-specific modulation, customized to tumor heterogeneity and disease progression.

In conclusion, autophagy is fundamental to ICD, facilitating ATP secretion and regulating other DAMPs to provoke antitumor immunity. The molecular processes, based on lysosomal exocytosis and ER stress signaling, highlight its therapeutic potential. Targeting autophagy-dependent DAMP release can improve ICD effectiveness, overcome immunosuppressive TME barriers, and synergize with immunotherapies, facilitating the development of advanced cancer treatments.

## Nanomaterials for modulating autophagy and ICD

Nanomaterials have revolutionized cancer treatment by offering precise, multifunctional platforms that concurrently affect complex biological processes such as autophagy and ICD [152, 153]. Their unique physicochemical properties, such as high surface area, adjustable dimensions, and stimuli-responsive behavior, enable targeted delivery, controlled release, and synergistic therapeutic effects, making them ideal for tackling the issues of autophagy dysregulation and improving the immunogenicity of ICD [154, 155].

Beyond direct induction of immunogenic cell death, nanomaterials can profoundly reshape antitumor immunity by reprogramming dysfunctional dendritic cells, enhancing antigen capture, and promoting sustained immune activation through multimodal interactions. Recent nanosponge- and catalytic nanoplatform-based strategies have demonstrated effective suppression of tumor progression and metastasis by integrating antigen sequestration, immune checkpoint regulation, and remote or stimuli-responsive immune modulation [156–159].

Importantly, ICD is characterized not just by molecular stress responses but by functional immunological activation. Established general principles define bona fide ICD as a regulated form of cell death that triggers an adaptive antitumor immune response in immunocompetent hosts. This process necessitates both antigenicity, which refers to the presence of tumor-associated antigens, and adjuvanticity, characterized by the release of immunostimulatory damage-associated molecular patterns (DAMPs), such as surface-exposed calreticulin (ecto-CRT), extracellular ATP, and HMGB1. These signals must ultimately facilitate dendritic cell maturation, antigen cross-presentation, cytotoxic T-cell priming, and observable in vivo immunogenicity, including vaccination-rechallenge protection or therapeutic synergy with immune checkpoint inhibition [160, 161].

Accordingly, the identification of isolated ICD markers, such as CRT exposure, ATP or HMGB1 release, or reactive oxygen species (ROS) generation, should be considered necessary yet inadequate for confirming functional ICD, as these characteristics may also result from immunogenic stress without triggering prolonged adaptive immunity [162]. This review differentiates between ICD-associated immunogenic stress, predominantly evidenced by in vitro or incomplete biomarker data, and functionally verified ICD, which is substantiated by downstream immune activation and/or in vivo validation.

Notably, the influence of nanomaterials on functional ICD or the predominant activation of cytoprotective autophagy is significantly context-dependent and determined by critical design and biological factors. The size of particles and their intracellular positioning affect the intensity and subcellular source of stress signals, whereas surface chemistry and coatings (such as PEGylation, targeting ligands, or biomimetic membranes) regulate cellular absorption, immune response, and antigen presentation. In addition, dose and exposure kinetics determine whether autophagy functions as an adaptive survival mechanism or is overwhelmed to favor pro-death signaling and ICD. Tumor type, basal autophagic flux, cancer stem cell reliance, and tumor microenvironmental characteristics, including hypoxia and acidity, significantly influence these outcomes.

It is worthy to mention that while ROS are central mediators of nanomaterial-induced stress, they do not drive ICD through a simple linear pathway. ROS generation can activate multiple unfolded protein response (UPR) sensors in the ER, including PERK, IRE1, and ATF6, which are differentially engaged depending on the type, localization, and surface properties of the nanomaterial. PERK activation leads to eIF2α phosphorylation and ATF4/CHOP induction, promoting CRT exposure and immunogenic cell death [163]. IRE1 triggers XBP1 splicing and JNK signaling, influencing autophagy and cytokine release, while ATF6 translocation upregulates ER chaperones that modulate cellular homeostasis and immune signaling. Distinct nanomaterials may preferentially engage specific UPR branches, shaping the balance between cytoprotective autophagy and pro-death ICD [164]. Recognizing this complexity enables rational nanomaterial design to optimize ICD while minimizing unintended adaptive responses.

Within this framework, nanomaterials (typically 1–100 nm) offer unique opportunities to regulate ICD and autophagy in a coordinated and context-dependent manner [165, 166]. Diverse nanoplatforms, including metallic, polymeric, lipid-based, and carbon-based systems, have been engineered to deliver ICD inducers, autophagy modulators, or combination payloads, each providing distinct advantages in terms of biocompatibility, targeting specificity, and immune modulation. In the following sections, we summarize recent nanomaterial-based strategies, critically evaluating the strength of ICD and autophagy evidence reported for each platform while highlighting design principles that favor robust and translatable antitumor immune responses.

### Metal-based nanoparticles

Metallic nanoparticles, including gold (AuNPs), silver (AgNPs), and iron oxide nanoparticles (IONPs), have become pivotal tools in cancer therapy owing to their distinctive physicochemical characteristics, such as plasmonic resonance, magnetic responsiveness, and the ability to generate ROS [167, 168]. These properties enable precise regulation of programmed cell death (PCD) pathways, including autophagy and ICD, which are crucial for overcoming tumor resistance and augmenting antitumor immunity. Metallic nanoparticles attain tumor-specific delivery and reduce off-target toxicity by either passive targeting through the enhanced permeability and retention (EPR) effect or active targeting by surface functionalization with ligands such as antibodies or peptides [169, 170].

**Fig. 4:**
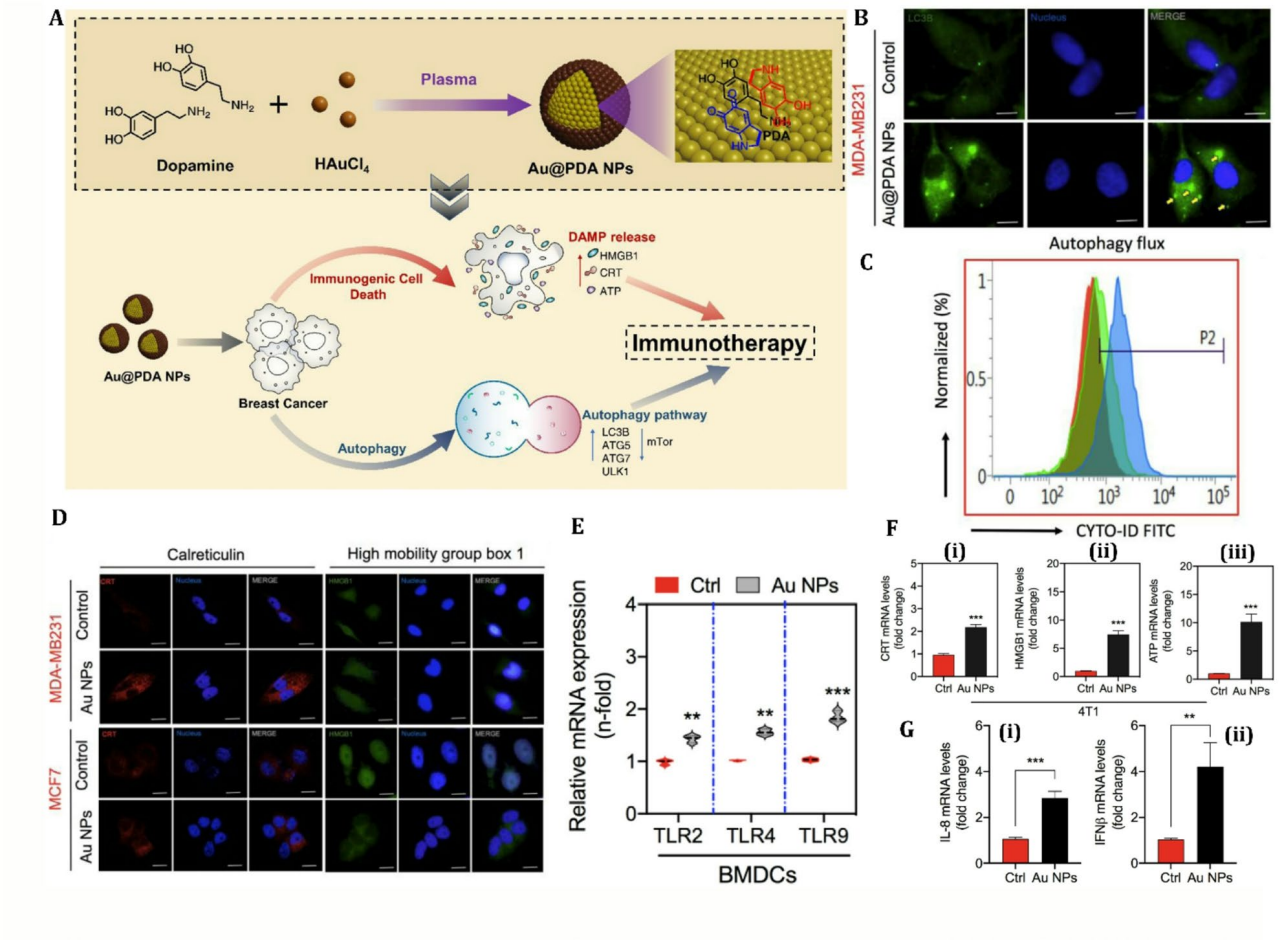
Overview of plasma-synthesized Au@PDA nanoparticles (NPs) and their immunomodulatory effects on breast cancer cells. **(A)** Schematic illustration: Top-cold plasma-assisted one-step synthesis of Au@PDA NPs; Bottom-proposed mechanism of autophagy and immunogenic cell death (ICD) induction upon treatment. **(B)** Au@PDA NPs trigger autophagy via mitochondrial damage accumulation in breast cancer cells, as visualized by LC3B immunofluorescence in MDA-MB-231 cells treated with 100 µM NPs. **(C)** Quantification of autophagic flux using CYTO-ID staining in MDA-MB-231 cells exposed to 20 µM and 100 µM Au@PDA for 48 h, analyzed by flow cytometry. **(D)** ICD-associated DAMP release induced by Au@PDA NPs: immunofluorescence imaging of CRT and HMGB1 in MDA-MB-231 and MCF7 cells treated with 100 µM Au@PDA. **(E)** Dendritic cell (DC) activation via co-culture with Au@PDA-treated cancer cells, assessed by TLR2, TLR4, and TLR9 mRNA levels in BMDCs after 48 h. **(F)** Gene expression analysis of CRT, HMGB1, and ATP in 4T1 murine breast cancer cells post-treatment with 100 µM Au@PDA; β-actin served as the reference gene. **(G)** Induction of pro-inflammatory cytokines IL-8 and IFN-β in MDA-MB-231 and MCF7 cells following Au@PDA exposure, indicating ICD-related immune activation. Reprinted from [176] with permission from Elsevier.

Gold nanoparticles (AuNPs) are distinguished for their plasmonic characteristics, facilitating photothermal therapy (PTT) and photodynamic therapy (PDT), as well as their capacity to regulate autophagy and ICD via specific signaling pathways [171–173]. Near-infrared photothermal therapy (NIR-PTT) using anti-EGFR (Epidermal Growth Factor Receptor) antibody-conjugated gold nanorods (anti-EGFR-GNs) could inhibit the AKT/mTOR pathway, a negative regulator of autophagy, resulting in autophagosome accumulation and autophagic cell death in TNBC models [174]. This process entails the activation of ATGs, such as LC3 and Beclin-1, thereby facilitating lysosomal breakdown [174]. AuNPs can promote ICD hallmarks by activating ER stress, leading to surface calreticulin (CRT) exposure and release of DAMPs, including HMGB1 and ATP. As an example, a study investigated the potential of AuNPs as radiosensitizers to enhance radiation-induced ICD in glioblastoma treatment [175]. The results show that the combination of radiotherapy (RT) with AuNPs elicits a more potent ICD impact on glioblastoma cells than RT alone. The combination of AuNPs and RT released key DAMPs (HMGB1, CRT, and ATP) and markedly increased the bone marrow-derived DC maturation, indicating functionally validated ICD [175]. In radiotherapy, AuNPs increase effectiveness by inhibiting protective autophagy and DNA repair mechanisms. Similarly, another research work explored the use of plasma-synthesized polydopamine-coated gold nanoparticles (Au@PDA NPs) to induce autophagy-dependent ICD in breast cancer cells, specifically MCF7, MDA-MB-231, and 4T1 cell lines [176]. The Au@PDA NPs, prepared via a plasma-liquid interaction method, exhibit high selectivity for cancer cells, triggering autophagy and the release of DAMPs such as CRT and HMGB1, which enhance antitumor immunity (Fig. 4). The study demonstrated that these nanoparticles inhibit cell proliferation, promote autophagic flux, and upregulate ICD markers. Using a 4T1 murine model, in vivo experiments demonstrated that Au@PDA NPs can reduce tumor growth and induce systemic anticancer immunity, also representing functionally validated ICD [176].

AuNPs modulate cellular autophagy by disrupting autophagic flux, mainly via lysosomal alkalinization, which hinders the fusion and degradation of autophagosomes and lysosomes [177]. Larger AuNPs are quickly internalized, resulting in lysosomal enlargement and the accumulation of autophagic vesicles, without directly triggering autophagy [178]. For instance, Au@Cu2-xSe nanoparticles were shown to alkalinize lysosomes, block autophagic flux, and inhibit DNA repair protein Rad51, sensitizing glioblastoma cells to radiation [179]. This disruption of autophagic flux and lysosomal dysfunction may induce cellular stress, potentially triggering ER stress and DAMP release, representing ICD-associated immunogenic stress (in vitro biomarkers only).

In addition to lysosomal effects, AuNPs influence immune cells, particularly macrophages, by modifying lysosome activity and membrane permeability, diminishing autophagic flux, and inhibiting M2-type polarization of TAMs. A research group synthesized polyethylene glycol-modified gold nanoparticles (PEG-AuNPs) that used these characteristics, showing diminished LC3 recycling and suppressed M2 polarization in TAMs, as validated by co-culture experiments utilizing autophagy inducers (ranibizumab) and inhibitors (chloroquine and Atg5 siRNA) [180]. The PEG-AuNPs facilitated DC maturation and elevated the ratios of CD4^+^ and CD8^+^ T-cells, hence enhancing antitumor immune responses [180]. The inhibition of autophagy in TAMs may facilitate the release of proinflammatory signals, potentially contributing to an immunogenic microenvironment conducive to ICD.

Moreover, AuNPs can lead to oxidative stress, hence enhancing autophagy activation. Researchers demonstrated that AuNPs in MRC-5 lung fibroblasts enhanced the expression of autophagy markers (ATG7, LC3-I) and oxidative stress indicators (MDA, PRP, OSRI), indicating that autophagy functions as a protective mechanism against oxidative stress [181]. This oxidative stress and autophagic activation may promote ER stress, a known trigger for DAMP release and ICD, necessitating further investigation. In another study, it was revealed that perovskite-cored gold-shelled nanoparticles specially eradicated tumor cells by impairing mitochondrial membrane potential and triggering autophagy at certain doses [182]. Such mitochondrial stress could enhance cellular immunogenicity by facilitating DAMP release, aligning with ICD mechanisms. Dhandapani et al. similarly showed that biosynthesized AuNPs produced ROS, affected mitochondrial membranes, and inhibited autophagic flow in gastric cancer cells, hence inducing cell death [183]. Surface-modified AuNPs can further regulate autophagy; for example, it was shown that 3-methyladenine-loaded AuNPs overcame radiotherapy resistance in cervical cancer by inhibiting autophagy [184]. By blocking protective autophagy, these AuNPs may enhance cellular stress, potentially promoting ICD in combination with radiotherapy. In another work, researchers have developed an internalizing-RGD (iRGD)-conjugated AuNP system that carries siRNA targeted to CDK7 (Cyclin-Dependent Kinase 7) to activate the antitumor immune response [185]. The system showed good tumor targeting performance and photothermal effects, inducing tumor cell necroptosis and improving the immunosuppressive microenvironment. This iRGD-conjugated AuNP/siCDK7 system could be a potential treatment strategy for lung adenocarcinoma. Xie et al. developed a furin-responsive platform delivering doxorubicin and hydroxychloroquine, inhibiting autophagy, resensitizing tumor cells to chemotherapy, and repolarizing TAMs to an M1 phenotype [186]. This modulation of autophagy and TAM polarization likely enhances the immunogenic TME, facilitating ICD.

A study demonstrated that AuNPs activate innate immune signaling pathways in a size-dependent manner, eliciting inflammatory responses and enhancing the desired immune response for effective immunotherapy [187]. Ultrasmall AuNPs activate the NLRP3 inflammasome, facilitating the maturation of caspase-1 and the production of interleukin-1β. Larger AuNPs activate the NF-κB signaling pathway. Au4.5 nanoparticles activate the NLRP3 inflammasome, leading to significant ROS production and the targeting of autophagy protein LC3, which is essential for the inhibition of NLRP3 inflammasome activation. Au4.5 nanoparticles serve as vaccine adjuvants, promoting ovalbumin-specific antibody production through an NLRP3-dependent mechanism [187]. The interplay between autophagy modulation and inflammasome activation may amplify DAMP release, further promoting ICD and antitumor immunity.

AuNPs cooperate with ICIs such as anti-PD-1 or anti-CTLA-4 by generating immunogenic TMEs. For instance, AuNPs targeted with anti-PD-L1 antibody passively target the glioma site, forming in situ aggregates that enhance the accumulation of doxorubicin and hydroxychloroquine [188]. This procedure inhibits autophagy, resensitizing glioma cells to DOX and inhibiting autophagy-related vasculogenic mimicry by glioma stem cells. In vivo studies also showed that D&H-A-A&C has a promising anti-glioma effect, and co-treatment with anti-PD-L1 antibody neutralizes the immunosuppressed glioma microenvironment [188]. By inhibiting autophagy and enhancing immunogenic signals, these AuNPs likely promote ICD, amplifying the efficacy of ICI therapy.

Overall, AuNPs induce autophagy by processes including lysosomal alkalinization, mitochondrial stress, and ROS generation, which all lead to cellular stress and DAMP release, key prerequisites for ICD. These results establish AuNPs as adaptable agents for cancer immunotherapy by linking autophagy and ICD with potential for synergistic treatments.

Silver nanoparticles (AgNPs) are recognized for their potent antimicrobial and anticancer properties, primarily inducing autophagy and ICD via mechanisms such as mitochondrial stress, ROS generation, and ER stress [189, 190]. They initiate mitophagy, a selective autophagic process, through the PINK1/PARKIN (PTEN-induced putative kinase 1/ Parkin RBR E3 ubiquitin ligase) pathway, which directs the degradation of damaged mitochondria [191, 192]. In A549 lung cancer cells, AgNPs decrease mitochondrial membrane potential, upregulating PINK1 and PARKIN, leading to mitophagosome formation [193]. Ultimately, the mitophagy-lysosomal pathway was activated, resulting in the apoptosis of tumor cells. This process promotes apoptosis and releases HMGB1, a key DAMP, that enhances ICD. AgNPs also can induce calreticulin, which further activates ICD and PCD pathways. As an example, a study investigated the effect of beta-D-glucose-reduced AgNPs (AgNPs-G) on breast cancer cells, demonstrating that AgNPs-G induces cell death in a dose-dependent manner in breast cancer cell lines and exhibits antiproliferative effects by disrupting the cell cycle [194]. Treatment with AgNPs-G promotes calreticulin exposure and the release of HMGB1, ATP, HSP70, and HSP90. Prophylactic immunization with AgNPs-G could not stop tumor formation in vivo; however, it did reduce tumor weight and improve survival rates in mice [194]. In contrast, a study conducted by Garcia et al. reported that AgNPs had anti-proliferative and anti-adhesion effects on CT26 colon carcinoma and MCA205 fibrosarcoma cells in vitro. However, the researchers did not identify any protective immune response or reduction in tumor growth in vivo. This finding suggests that the cell death was through non-immunogenic mechanisms, such as apoptosis and non-canonical autophagy, indicating ICD-associated immunogenic stress [195].

AgNPs exhibit significant cytotoxicity against cancer cells, making them potential anticancer agents [196]. Studies have shown that AgNPs promote cytoprotective autophagy in tumor cells, possibly diminishing the effectiveness of drug treatments or radiotherapy [197]. This autophagy is activated through the PtdIns3K (Phosphatidylinositol 3-Kinase) pathway, increasing autophagosome formation without impairing lysosomal function, thus serving as a pro-survival mechanism [197, 198]. The combination of AgNPs with autophagy inhibitors such as wortmannin or ATG5 knockdown increases their therapeutic efficacy [198]. A research team subsequently demonstrated that AgNPs enhance autophagy by dephosphorylating TFEB at serine-142 and serine-211, hence augmenting its nuclear translocation and the expression of ATGs [199]. In glioma cells, AgNPs enhance radiosensitivity while inducing protective autophagy via ERK ( Extracellular Signal-Regulated Kinase) and JNK (c-Jun N-terminal kinase pathway) pathways; inhibiting this autophagy with 3-MA elevates ROS, boosting radiosensitization [200]. Silver ions from AgNPs cause mitochondrial damage, increasing ROS and triggering autophagy [201]. For instance, UV-induced Ag@ZnO nanoparticles damaged the Golgi, leading to excessive autophagy and melanocyte apoptosis [202]. Polymer-coated hybrid AgNPs, such as gold–silver nanoparticles with polydopamine, induce autophagy and mitochondrial damage while mediating photothermal tumor killing [203]. Silver ion release or internalization through phagocytosis is the main cause of AgNP cytotoxicity, which has the potential to disrupt organelles and induce necrosis [204]. Smaller particle sizes, co-administration with autophagy inhibitors, or targeted delivery can reduce toxicity while enhancing efficacy [198].

**Fig. 5:**
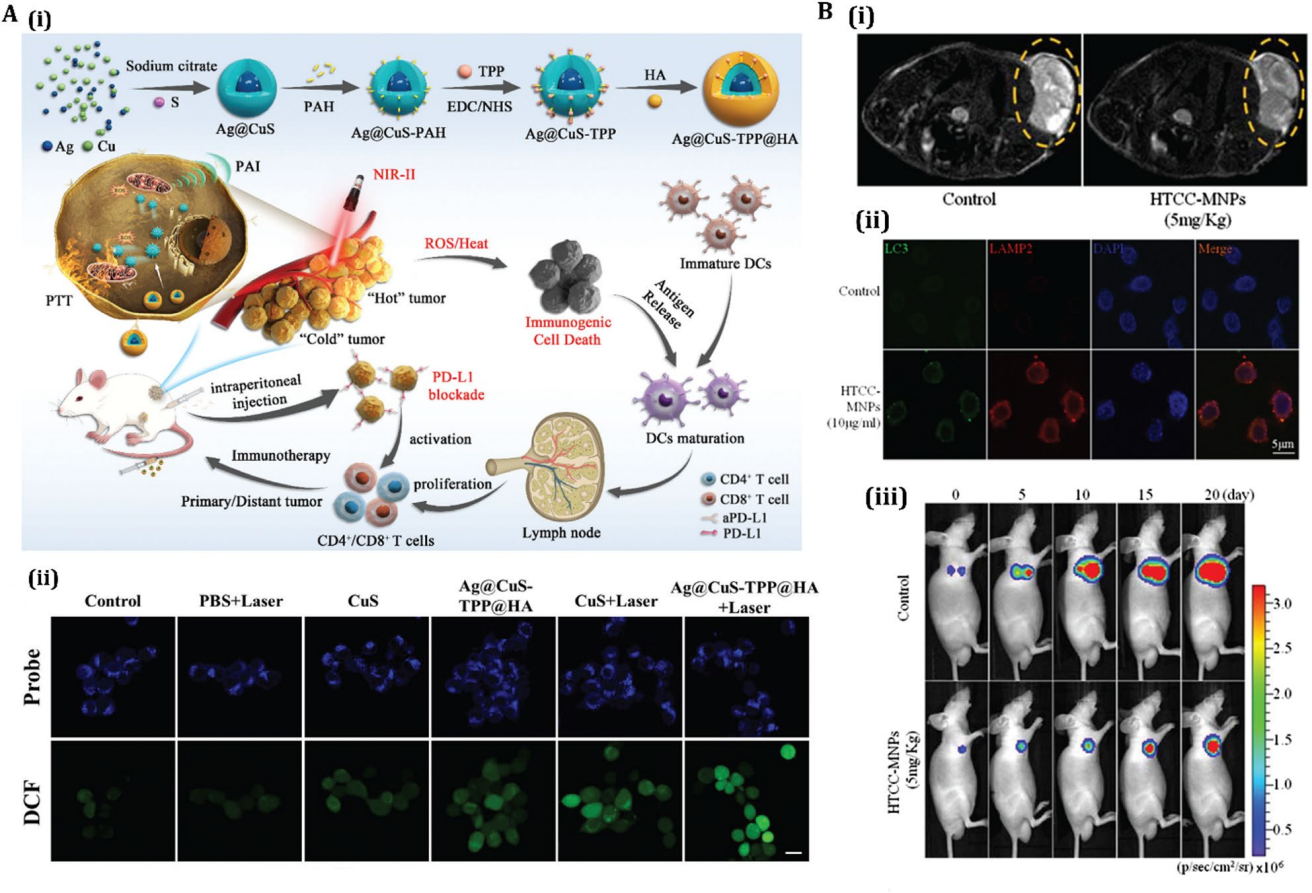
Evaluation of AgNPs and IONPs for modulation of autophagy and ICD: **(A)** Schematic and mechanistic overview of Ag@CuS-TPP@HA nanoparticles: **(i)** Illustration of the nanoparticle synthesis and therapeutic strategy, highlighting how ROS generation and photothermal therapy (PTT) induce immunogenic cell death (ICD), enhance cytotoxic T cell infiltration, elicit systemic antitumor immunity, and synergize with immune checkpoint blockade (ICB) to suppress tumor progression. **(ii)** Confocal imaging of intracellular ROS levels under various treatments, visualized through dual fluorescence channels. Reprinted from [206] with permission from Wiley. **(B)** In vivo and in vitro assessment of quaternized chitosan/alginate-Fe_3_O_4_ magnetic nanoparticles (HTCC-MNPs): **(i)** MRI imaging 24 h post-injection shows nanoparticle accumulation at the tumor site in mice treated with HTCC-MNPs versus PBS controls, reflected by signal intensity reduction. **(ii)** Immunofluorescence imaging reveals autophagic activity in SGC7901/ADR cells, shown by LC3 and LAMP2 colocalization following HTCC-MNP treatment. **(iii)** Tumor progression tracking via bioluminescence imaging (BLI) in mice implanted with SGC7901/ADR+Fluc cells, with imaging conducted on days 0, 5, 10, 15, and 20 after HTCC-MNP administration. Ex vivo data confirm the inhibitory effect of the treatment. Reprinted from [213] with permission from American Scientific Publishers

AgNPs’ potential toxicity, including organ accumulation and oxidative stress in healthy tissues, remains a concern. Strategies like polymerization or hybrid designs with polymeric shells are being explored to enhance biocompatibility. For instance, a study has found that a combination of shikonin (SHK) and chitosan silver nanoparticles (Chi-Ag NPs) can induce necroptosis in 4T1 breast cancer cells, effectively triggering ICD [205]. The researchers developed an MUC1 aptamer-targeted nanocomplex (MUC1@Chi-Ag@CPB@SHK) to co-deliver SHK and Chi-Ag NPs, which increased the concentration of MUC1@ACS at the tumor site by 6.02-fold. This upregulated the expression of RIPK3 (Receptor-Interacting Protein Kinase 3), p-RIPK3 (phosphorylated RIPK3), and tetrameric MLKL (Mixed Lineage Kinase Domain-Like protein), triggering ICD. The study also showed that DCs enhanced the infiltration of CD8^+^ and CD4^+^ T cells in tumors, inhibiting regulatory T cells, thus representing functionally validated ICD [205]. Bai et al. also introduced AgNPs modified with copper (II) sulfide and targeted with triphenylphosphonium bromide and hyaluronic acid (Ag@CuS-TPP@HA), designed for dual-targeting cancer cells and mitochondria to enhance antitumor therapy [206]. The nanoparticles, surface-modified with TPP and HA, exhibit strong near-infrared II photoacoustic imaging and photothermal effects. They catalyze Fenton-like reactions, producing ROS that induce ICD and release tumor-associated antigens (Fig. 5**A**). In vivo experiments show complete primary tumor ablation and significant tumor growth inhibition, indicating potential for precise cancer diagnosis and effective immunotherapy [206]. By modulating autophagy to enhance ICD, these approaches can overcome the protective effects of autophagy, amplifying the immunogenic potential of AgNP-induced cell death. This synergy between autophagy inhibition and ICD induction holds promise for improving the therapeutic efficacy of AgNPs in cancer treatment.

Iron oxide nanoparticles (IONPs) are also increasingly used for their multifaceted roles in cancer therapy, utilizing their magnetic properties, biocompatibility, and ability to induce autophagy and ICD through mechanisms such as oxidative stress, ROS generation, and mitochondrial dysfunction [207, 208]. IONPs stimulate autophagy by activating pathways like AMPK/mTOR, promoting autophagosome formation and lysosomal degradation, often as a cytoprotective response to oxidative damage [209]. In MCF-7 breast cancer cells, IONPs elevate ROS levels, upregulating LC3-II and Beclin-1, leading to autophagic flux and subsequent apoptosis [210]. This process releases DAMPs, such as HMGB1 and ATP, enhancing ICD and stimulating immune responses via DC activation and T-cell infiltration. IONPs, such as Fe_2_O_3_@DMSA, use magnetic and ROS-generating properties to induce pro-death autophagy and ICD. They facilitate Fenton reactions, producing ROS that disrupt autophagosome-lysosome fusion, leading to autophagic cell death in HCC cells [211]. This ROS-mediated stress also triggers ER stress, exposing calreticulin and releasing DAMPs like HMGB1 and ATP, amplifying ICD. Polyethyleneimine-modified Fe_3_O_4_ nanoparticles (PEI-MNPs) upregulate ATG7, promoting autophagosome formation, and generate ROS via Fenton-like reactions, enhancing autophagy and PCD [212].

The interplay between autophagy and ICD is critical, as autophagy can modulate ICD outcomes. While pro-death autophagy, as induced by Fe_2_O_3_@DMSA, enhances ICD by promoting DAMP release and immune activation, cytoprotective autophagy may reduce ICD efficacy by aiding tumor cell survival, thereby limiting DAMP exposure and immune stimulation [214, 215]. For example, it was shown that the inhibition of autophagy using drugs such as chloroquine (CQ) or 3-methyladenine (3-MA) can enhance ICD by redirecting cellular responses towards apoptosis, hence increasing the release of DAMPs and immunogenicity. A study showed that the combination of CQ or 3-MA with IONP-mediated photothermal treatment (PTT) enhanced cytotoxicity, resulting in an increase in tumor inhibition from 43.26% to 68.56% in the IONP/CQ group, with immunohistochemistry analysis indicating greater LC3 expression and apoptosis [216]. This evidence suggests the inhibition of autophagy might synergize IONP-induced ICD to amplify anticancer effects.

Furthermore, IONPs, such as Fe_2_O_3_ NPs, demonstrate diagnostic potential, with superparamagnetic iron oxide nanoparticles (SPIONs) recognized as FDA-approved MRI contrast agents. Zhang et al. synthesized Fe_3_O_4_ NO· NPs (NO· radical-conjugated Fe_3_O_4_ NPs; NO·, nitric oxide free radical) that improve T1- and T2-weighted imaging through the utilization of autophagy-induced reactive substances. This advancement offers a technique for visualizing autophagic flux in vivo, potentially guiding ICD-based therapeutic approaches [217]. However, IONP toxicity, driven by ROS, mitochondrial disruption, and DNA damage, depends on factors like particle size (optimally 10–100 nm) and surface coatings. To address toxicity, researchers are exploring surface functionalization, as seen with quaternized chitosan/alginate-Fe_3_O_4_ magnetic nanoparticles (HTCC-MNPs), which enhance chemosensitization in multidrug-resistant (MDR) gastric carcinoma [213]. The nanoparticles induced autophagy and apoptosis in a dose- and time-dependent manner, mediated by increased ROS generation, mitochondrial dysfunction, and caspase-3 activation (Fig. 5**B**). In vivo studies using MRI and bioluminescence imaging showed significant tumor growth inhibition in SGC7901/ADR tumor-bearing mice, attributed to the nanoparticles’ tumor-targeting ability via the EPR effect [213].

Moreover, IONP-based PTT has been shown to enhance anti-CTLA-4 immunotherapy by using IONP-PTT to deplete tumor-associated regulatory T cells (Tregs) in a 4T1 murine breast tumor model [218]. The study demonstrates that systemically administered IONPs accumulate in tumor tissues, enabling near-infrared light-triggered hyperthermia that preferentially eliminates immunosuppressive Tregs, thereby enhancing CD8^+^ T-cell activation. Sequential PTT, combined with anti-CTLA-4 therapy, significantly inhibits primary and distal tumor growth, reduces lung metastasis, and generates memory T cells to prevent tumor recurrence, representing functionally validated ICD [218]. By carefully balancing autophagy induction and inhibition, IONPs can be tailored to maximize ICD, enhancing immune activation while minimizing tumor survival. Advances in surface modification and combination therapies with autophagy inhibitors continue to optimize IONP safety and efficacy, positioning them as promising tools for precise cancer diagnosis, immunotherapy, and synergistic ICD-based treatments.

### Polymer-based nanoparticles

Polymer-based nanoparticles have emerged as versatile platforms in cancer therapy due to their tunable physicochemical properties, biocompatibility, and ability to modulate autophagy and ICD for enhanced therapeutic outcomes [219, 220]. These nanoparticles, composed of natural or synthetic polymers such as chitosan, polyethyleneimine (PEI), poly(lactic-co-glycolic acid) (PLGA), and polyethylene glycol (PEG), offer precise control over size, surface charge, and drug encapsulation, enabling targeted delivery and controlled release [221, 222]. By integrating autophagy modulators and ICD inducers, polymers can regulate cellular processes to overcome tumor resistance, increase immune responses, and improve cancer diagnostics and treatment efficacy [223–225].

More recently, researchers offered a new strategy that involves creating tailor-made autophagy cascade amplification polymeric nanoparticles STF@AHPPE, which are grafted onto hyaluronic acid and loaded with autophagy inducer STF-62247 [226]. These nanoparticles provoke intense cytotoxic autophagy and robust ICD efficacy, representing functionally validated ICD, offering an innovative approach to combining tumor chemo-immunotherapy with autophagy induction [226]. In another work, in order to enhance the efficiency of chemotherapeutic agents, a research group developed a ROS-responsive self-immolative polymer (R–SIP) to enhance DOX’s immunogenicity. When treated with 4T1 cancer cells, DR-SIP induces ER-associated ICD and DC maturation, inhibiting tumor growth in mice compared to DOX alone [227].

Similarly, to enhance antitumor immunity, a self-delivery micelle was formulated to activate ICD and autophagic cell death simultaneously [228]. This micelle targets αvβ3 on tumor cells, triggering ICD and releasing associated molecular patterns. The combination of oxaliplatin (chemotherapeutic agent) and SMER28 (autophagy activator) suppresses tumor proliferation and activates antitumor immune responses, offering a viable option for antitumor immunotherapy [228]. In another study, a dendritic polymer-functionalized nanomedicine was developed that effectively reduces PD-L1 abundance in colon cancer cells [229]. This treatment promoted AMPK phosphorylation, leading to PD-L1 degradation and T cell activation. The polymer-based NPs induced ICD and enhanced anticancer immunity. Combining this with PD-1 blockade enhances cytotoxic T lymphocyte activity and inhibits tumor growth without side effects [229]. A phenolic nanoadjuvant was developed to modulate DC function and improve cancer immunotherapy. The nanoadjuvant was developed by self-assembly of the sonosensitizer polymer (PEG-b-IR), GSH inhibitor (sabutoclax), Mn^2+^, and TME acidic sensitive phenolic polymer (PEG-b-Pho) via metal–phenolic coordination [230]. This platform activates the cGAS-STING pathway, enhancing DC maturation and sensitizing tumors to PD-L1 checkpoint blockade immunotherapy. This approach effectively inhibits distant tumor growth and restrains lung metastasis, potentially addressing the therapeutic limitation of insufficient antitumor immunity [230].

**Fig. 6:**
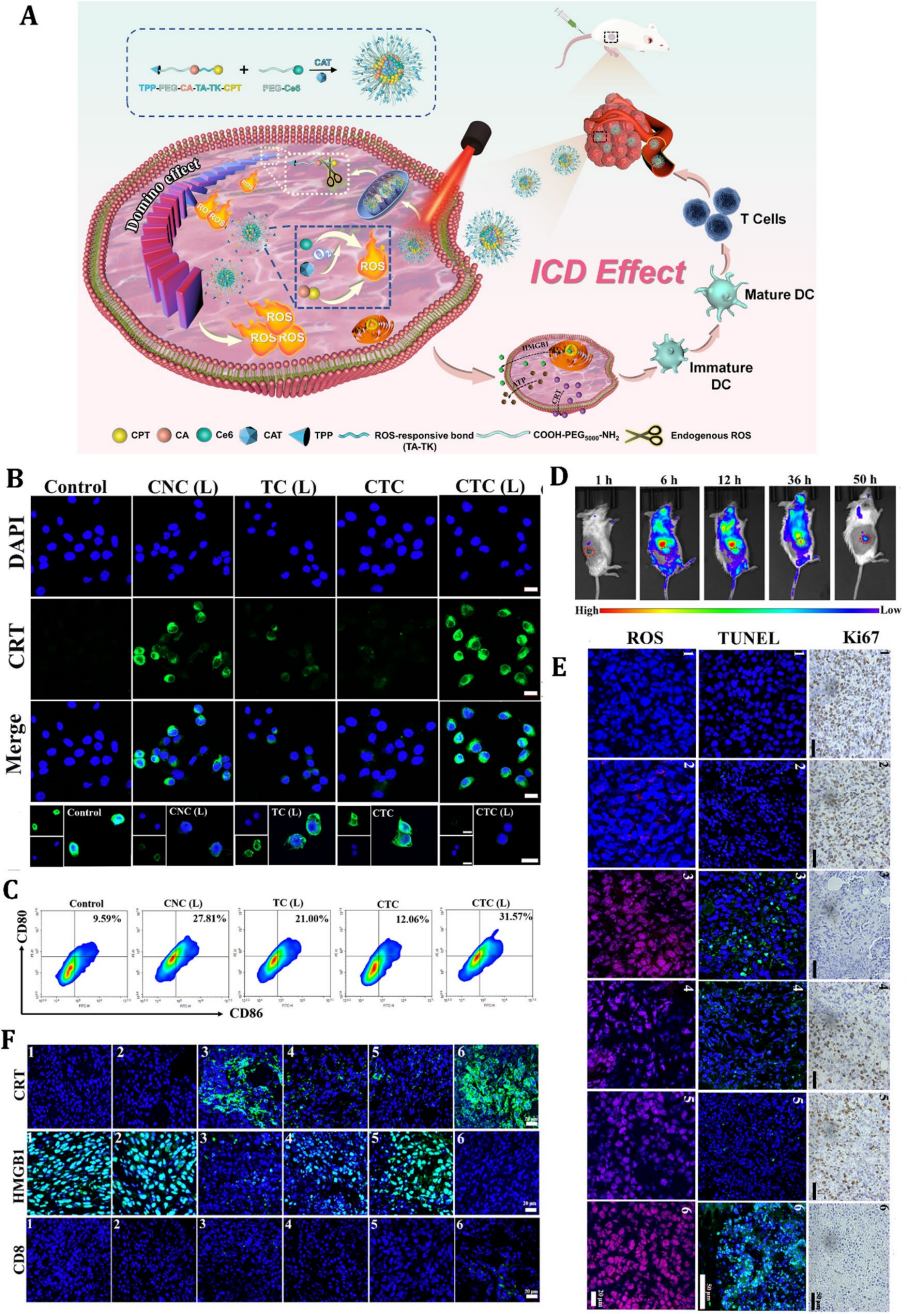
Evaluation of ROS-responsive, mitochondria-targeted polymeric micelles for enhanced ICD induction and antitumor immune activation. **(A)** Schematic representation of the synthesis and mechanism of action of ROS-responsive, triple-synergistic micelles designed for mitochondrial targeting. Upon cellular internalization, ROS-triggered micelle disassembly initiates a cascade effect, leading to robust immunogenic cell death (ICD). **(B)** Confocal laser scanning microscopy (CLSM) images of 4T1 cells treated with different formulations, showing calreticulin (CRT) exposure and HMGB1 release as markers of ICD. **(C)** Flow cytometric analysis of dendritic cell maturation (CD80^+^CD86^+^) after co-culture with pretreated 4T1 cells, demonstrating immune activation potential. **(D)** In vivo fluorescence imaging of 4T1 tumor-bearing mice at multiple time points following systemic administration of CTC micelles, showing biodistribution and tumor accumulation. **(E)** Immunohistochemical analysis of tumor sections on day 14 post-treatment, stained for Ki67 (proliferation marker), TUNEL (apoptosis), and ROS to evaluate therapeutic effects. **(F)** Immunofluorescence staining of tumor tissues to assess CRT and HMGB1 expression, along with infiltration of CD8^+^ cytotoxic T cells, indicating effective ICD and antitumor immune response in treated groups. Reprinted from [232] with permission from Elsevier.

Furthermore, a phenylboronic acid-modified polymeric nano-prodrug based on β-cyclodextrin and SHK has also been developed for tumor immunotherapy [231]. The nanoparticles show high tumor targeting and efficient cellular uptake, induced programmed cell death, and reduced immunosuppression. The introduction of chloroquine terminates autophagic flux, enhancing antigen exposure and reducing tumor burden and metastasis [231]. Researchers likewise introduced a ROS-responsive, mitochondria-targeted polymer micelle system (CTC micelles) designed to induce ICD for enhanced tumor immunotherapy [232]. These micelles, encapsulating catalase (CAT) and integrating camptothecin (CPT), chlorin e6 (Ce6), and triphenylphosphonium (TPP), use a triple-synergistic approach to amplify ROS production within tumor cell mitochondria (Fig. 6). CPT and CAT stimulate ROS generation, Ce6 enhances ROS under 660 nm laser irradiation via PDT, and CAT mitigates hypoxia, boosting PDT efficacy. The micelles target mitochondria, triggering a ROS domino effect that induces ICD, marked by CRT exposure, HMGB1, and ATP release, which promotes DC activation and CD8^+^ T-cell infiltration (Fig. 6). In vivo studies using a bilateral 4T1 tumor model demonstrate significant primary and distant tumor suppression via ICD-mediated immune responses, with transcriptomics revealing pathways like PI3K/AKT and reduced Foxp3 expression, alleviating immunosuppressive TMEs [232]. These findings highlight the versatility of polymer-based NPs in modulating autophagy and ICD to enhance cancer immunotherapy, offering innovative solutions to address tumor resistance and immune evasion.

### Lipid-based nanoparticles

Lipid-based nanoparticles (LNPs) have gained prominence as advanced platforms in cancer therapy due to their biocompatibility, structural versatility, and ability to modulate autophagy and ICD for enhanced therapeutic and diagnostic outcomes [233, 234]. Lipid nanoparticles (LNPs), including liposomes, solid lipid nanoparticles (SLNs), and nanostructured lipid carriers (NLCs), consist of lipids such as phospholipids, cholesterol, and triglycerides, providing distinct benefits such as elevated drug-loading capacity, tunable surface changes, and effective cellular absorption [235, 236]. These properties enable LNPs to deliver autophagy inducers, ICD activators, and imaging agents with precision, overcoming tumor resistance and amplifying antitumor immune responses [237].

LNPs modulate autophagy by delivering bioactive molecules, generating ROS, or disrupting cellular homeostasis. For instance, a liposomal nanosystem encapsulating copper peroxide nanodots (CPNs) and artemisinin (ART) was developed for autophagy-enhanced and ferroptosis-involved cancer cell death [238]. The CPNs self-supply H_2_O_2_ and Cu^2+^, while ART acts as an autophagy inducer, increasing intracellular iron levels and promoting cancer cell ferroptosis, suggesting ICD-associated immunogenic stress. Similarly, light-responsive nanoliposomes, encapsulated with IR780 dye and a bioactive chlorophyll-rich fraction of Anthocephalus cadamba (CfAc), have been found to induce cancer cell death through heat and ROS-mediated autophagy [239]. In both in vitro and in vivo conditions, these NIR light-activated nanoliposomes showed localized synergistic cancer cell death.

In a complementary approach, researchers proposed a combined tumor-therapeutic strategy for cancer treatment that utilizes nanosonosensitizers-augmented noninvasive sonodynamic therapy (SDT) along with autophagy inhibition [240]. The nanoliposomes co-encapsulate clinically approved sonosensitizers protoporphyrin IX and early-phase autophagy-blocking agent 3-methyladenine (3-MA). This strategy reduces cell resistance to oxidative stress and improves cancer-cell apoptosis, providing a proof-of-concept for ROS-resistant cancer treatment [240].

In contrast, LNPs can inhibit cytoprotective autophagy to enhance therapeutic efficacy. For example, a Prz-loaded liposome hybrid nanovesicle (Prz@LINV) system has been developed that enhances Prz bioavailability and significantly inhibits tumor cell autophagy. This can eventually lead to ICD and anticancer immune responses, while the system also modulates DCs, augmenting their capacity for antigen cross-presentation [241]. Another interesting study presented a liposome nanodrug co-encapsulated with doxycycline hydrochloride and chlorin e6 that induces autophagy inhibition and mitochondrial dysfunction, potentiating tumor photo-immunotherapy [242]. This combination generates ROS, elicits robust photodynamic therapy-induced ICD, and enhances photodynamic therapy-induced ICD. The nanodrugs improve cancer immunotherapy by combining Ce6-mediated PDT and Doxy-induced autophagy inhibition and mitochondrial dysfunction [242]. Researchers also offered a novel approach to cancer treatment by using mitochondrial-targeting liposomal nanoparticles (MLipRIR NPs) synthesized to encapsulate R162 and IR780 [243]. These nanoparticles disrupt the glutaminolysis pathway in mitochondria, causing downregulation of glutathione peroxidase (GPx). This process leads to apoptosis and lipid peroxide accumulation, triggering ICD. This therapy can activate antitumor immunity, suppressing both primary and distant tumors.

**Fig. 7:**
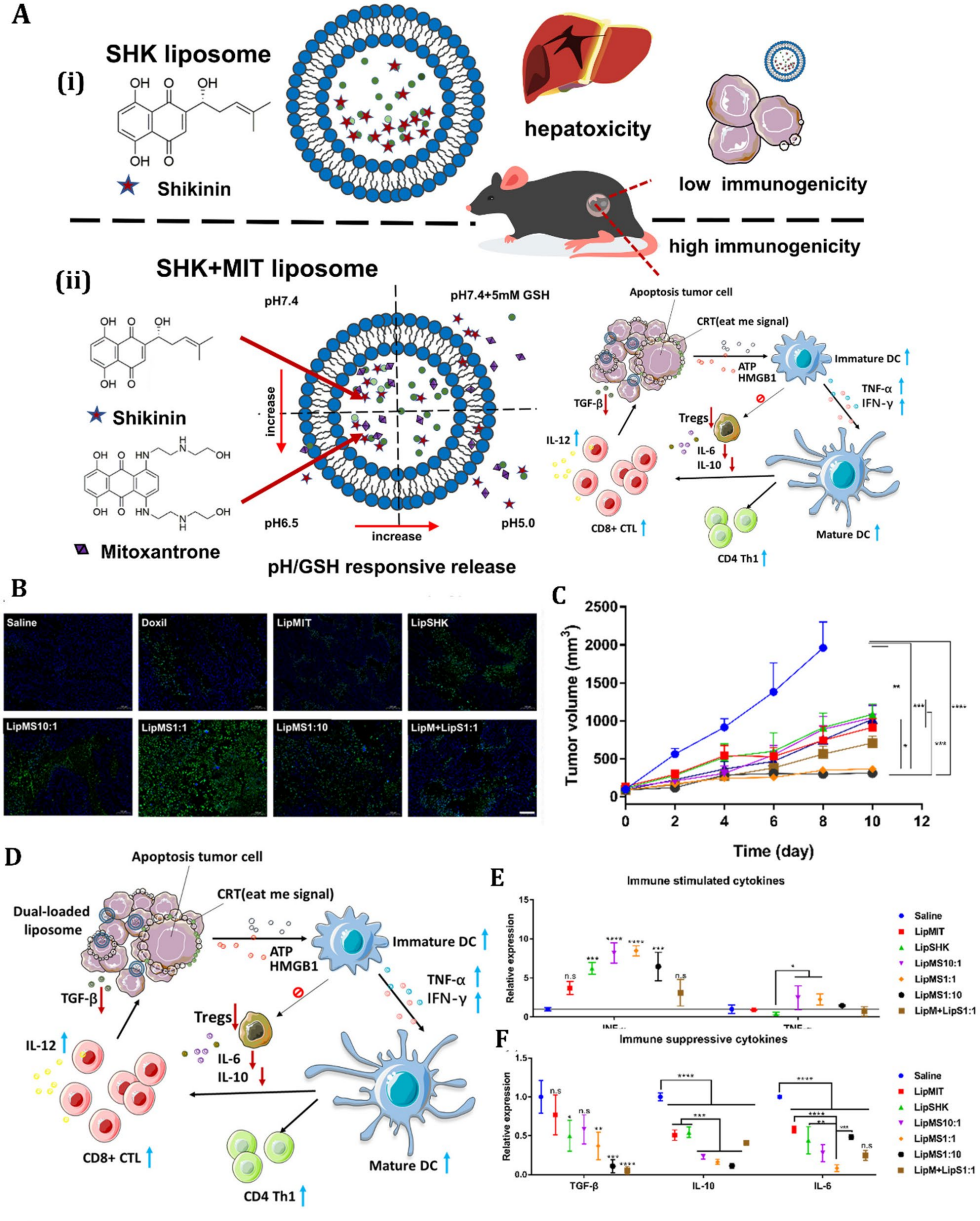
Dual-responsive SHK/MIT co-loaded liposomes for synergistic chemoimmunotherapy through enhanced ICD induction. **(A)** Schematic overview of copper-mediated liposomes co-encapsulating shikonin (SHK) and mitoxantrone (MIT): **(i)** SHK-loaded liposomes alone showed limited immunogenicity, and higher doses required for immunogenic cell death (ICD) induction posed hepatotoxicity risks. **(ii)** Co-delivery of SHK and MIT in liposomes with pH- and glutathione (GSH)-responsive release profiles enabled efficient tumor targeting, enhanced apoptosis, and robust induction of ICD and adaptive immunity. **(B)** TUNEL staining of tumor tissues illustrating increased apoptosis following treatment with dual-loaded liposomes. **(C)** Tumor growth curves showing the therapeutic efficacy of different formulations, with significant inhibition observed in the dual-loaded group. **(D)** Schematic illustration of the ICD process and activation of the adaptive immune response induced by SHK/MIT liposomes in melanoma. **(E)** Quantification of immune-stimulatory cytokines in tumor tissues, reflecting enhanced immune activation. **(F)** Analysis of immunosuppressive cytokines post-treatment, demonstrating the immunomodulatory effect of the liposomal formulation. Reprinted from [246] with permission from Elsevier.

Moreover, a liposome encapsulating SHK, a powerful ICD inducer, has been developed to elicit ICD in vivo at high dosage [244]. Nonetheless, it caused hepatotoxic consequences. To address this, SHK was co-encapsulated with the doxorubicin or anthracyclines mitoxantrone in liposomes, resulting in synergistic ICD effects and cytotoxicity against tumor cells. To determine the optimal synergistic ratio of SHK and anthracyclines, cytotoxicity and uptake experiments were conducted [244]. The antitumor effect was enhanced by the dual-loaded liposomes, which were safer than the single-loaded liposomes and stimulated a stronger immune response at lower dosages. Another study introduced a new life-prolonging therapy for pancreatic ductal adenocarcinoma (PDAC) using a silicasome, which is a lipid bilayer-coated mesoporous silica nanoparticle [245]. The silicasome improves PDAC survival through a chemo-immunotherapy response, triggering a cascade of events including autophagy inhibition, ER stress, ICD, and programmed death-ligand 1 (PD-L1) expression. This enhanced antitumor immunity is more robust than free or liposomal drugs like Onivyde [245]. A compensating strategy was implemented by incorporating ATP as a remote loading gradient into the liposome to encapsulate an autophagy inhibitor (hydroxychloroquine, HCQ). LipHCQa (HCQ-loaded liposomes with ATP) demonstrated remarkable antitumor efficacy without compromising immune response [246]. A systematic assessment of the optimum dose of combined LipSHK and LipHCQa indicated that autophagy inhibition at a suitable dosage was advantageous for maximizing ICD-mediated antitumor immunity (Fig. 7). Autophagy inhibitors have promising clinical applications in cancer immunotherapy, as proved in this study, which can restore a compromised ICD-based antitumor immune response [246]. Collectively, these advancements highlight the versatility of LNPs in modulating autophagy and ICD to address complex challenges in cancer therapy.

### Carbon-based nanoparticles

Carbon-based nanoparticles, including carbon nanotubes (CNTs), graphene oxide (GO), carbon dots (CDs), and fullerenes, have emerged as promising platforms in cancer therapy owing to their distinctive physicochemical properties, such as high surface area, adjustable functionalization, and superior optical and electrical characteristics [247, 248]. These properties enable carbon-based platforms to serve as versatile carriers for drugs, imaging agents, and bioactive molecules, facilitating precise modulation of autophagy and ICD to enhance therapeutic and diagnostic outcomes. By using their ability to generate ROS, target specific cellular pathways, and activate immune responses, nanoparticles offer innovative solutions to overcome tumor resistance and amplify antitumor immunity [249].

Carbon-based nanoparticles modulate autophagy by delivering bioactive molecules, inducing oxidative stress, or disrupting cellular homeostasis. For instance, it was shown that exposure to carbon black NPs induces inflammation and apoptosis in cardiomyocytes, increasing ROS and mitochondria fragmentation [250]. The study also found that carbon black NPs impair autophagy, leading to dysfunctional mitochondria and NF-κB activation. Another study evaluated the effects of single-walled carbon nanotubes (SWCNTs) on human bronchial epithelial cells [251]. The results show that SWCNTs decrease ATP production and cell viability and increase the expression of superoxide dismutase-1 and ROS generation. They also enhance the release of nitric oxide, IL-6, and IL-8, and upregulate ATGs [251]. The findings suggest that SWCNTs induce autophagic cell death through mitochondrial dysfunction and cytosolic damage.

**Fig. 8:**
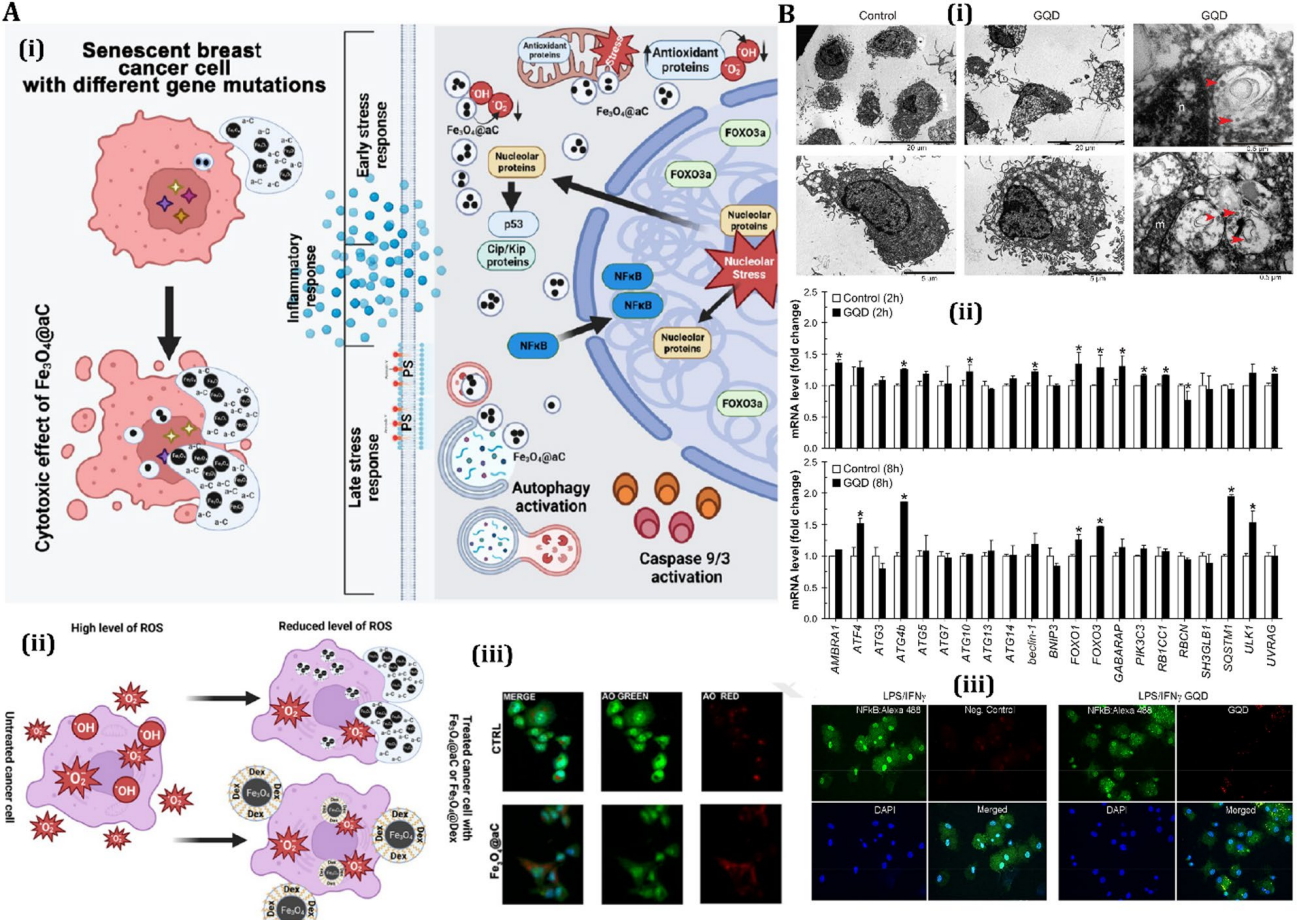
Reductive stress and ROS-mediated autophagy pathways induced by carbon-coated iron oxide and graphene-based nanomaterials. **(A)** Mechanistic effects of carbon-coated iron oxide nanoparticles (Fe₃O₄@aC) in drug-induced senescent breast cancer cells: **(i)** Schematic depiction of Fe₃O₄@aC-induced cytotoxicity in breast cancer cells with different mutation profiles. The cytotoxic effects are primarily mediated by reductive stress—characterized by decreased ROS levels and upregulation of antioxidant proteins including FOXO3a, SOD1, and GPX4. This state triggers downstream effects such as nucleolar stress (protein relocalization), activation of inflammatory signaling (NF-κB, IL-6, IL-8), cell cycle arrest (upregulation of p16, p21), autophagy induction (increased BECN1 and LC3B), and apoptosis via caspase 9 and caspase 3 activation and phosphatidylserine externalization. **(ii)** Graphical representation of reductive stress in Fe₃O₄@aC-treated breast cancer cells. **(iii)** Impact of Fe₃O₄@aC on cell cycle inhibitor expression and lysosomal activity, the latter visualized via acridine orange staining. Representative images include green, red, and merged channels. Reprinted from [252] with permission from American Chemical Society. **(B)** Autophagy and inflammatory modulation in dendritic cells (DCs) by graphene quantum dots (GQD): **(i)** Transmission electron microscopy (TEM) of GM-CSF/IL-4-induced DCs treated with GQD (200 µg/mL, 24 h) reveals multilamellar vesicles and autophagic compartments containing internalized GQDs and organelles (nucleus, mitochondria). **(ii)** Quantitative RT-PCR analysis of autophagy-associated gene expression in DCs after 2 and 8 h GQD exposure, indicating modulation of key transcriptional regulators. **(iii)** GQD exposure reduces ROS production and inhibits NF-κB nuclear translocation in DCs. NF-κB localization was assessed via epi-fluorescence microscopy, and relative fluorescence intensity ratios (nucleus/cytoplasm) were calculated. Representative images are provided. Reprinted from [254] with permission from Elsevier

In another notable study, researchers tested Fe_3_O_4_ NPs coated with dextran and glucosamine-based amorphous carbon coating against drug-induced senescent breast cancer cells (Fig. 8**A**) [252]. The results showed a decrease in ROS production, increased antioxidant proteins, and increased levels of proinflammatory and autophagic markers. The nanoplatforms also promoted reductive stress-mediated cytotoxicity in nonsenescent breast cancer cells [252].

Furthermore, carbon-based nanoparticles play a critical role in inducing ICD by releasing DAMPs. For example, a study examined the immunogenicity of graphene-induced hyperthermia (GIHT) in melanoma B16F10 cells, either alone or combined with radiotherapy [253]. In vitro tumor cell death exhibited apoptosis, which advanced to secondary necrosis. The simultaneous release of HSPs, HMGB1, and ATP was observed 24 h post combined treatment [253]. The research indicates that the concurrent release of several DAMPs could foster anti-tumor immunity against previously irradiated tumor cells, reducing tumor growth at the site of inoculation. Non-toxic doses of graphene quantum dots (GQD) inhibit proinflammatory and Th1 cytokines production and augment anti-inflammatory and Th2 cytokine production by human peripheral blood mononuclear cells [254]. GQD also impairs the differentiation and functionality of monocyte-derived DCs, diminishing their ability to promote T cell proliferation and cytotoxicity. DC treated with GQD potentiates Th2 polarization and induces suppressive regulatory T cells (Fig. 8**B**).

In a complementary approach, researchers have developed a targeted photosensitizer for PTT of solid tumors using the SWCNT-ANXA5 conjugate [255]. This treatment synergizes with checkpoint inhibition, causing a systemic anticancer immune response. In vitro ablation promoted cell death in a dose-dependent manner. In vivo tests were conducted using the orthotopic EMT6 breast tumor model in female balb/cJ mice. Intratumoral injection of the conjugate and lower PTT temperature improved therapeutic effects. When combined with checkpoint inhibition, the SWCNT-ANXA5-mediated PTT increased survival and 80% of mice survived for 100 days [255]. Overall, these studies highlight the multifaceted roles of carbon-based nanoparticles in modulating autophagy and ICD, offering innovative strategies for cancer therapy.

Table 1 summarizes some recent investigations on the role of different nanomaterials for modulation of autophagy and ICD.

**Table 1:** Recent Studies on Nanomaterials as Modulators of Autophagy and ICD in Cancer Therapy

Nanomaterial type	Formulation	Mechanism of action	Cancer model	Key outcomes	Level of ICD evidence	Ref.
Titanium dioxide NPs	TiO₂ NPs (30 and 100 nm)	Induces ROS-mediated autophagy (LC3, Beclin-1 upregulation) and ICD-associated DAMP release	RAW 264.7 macrophages	Reduced cell viability; enhanced autophagy and phagocytic modulation	ICD-associated immunogenic stress (in vitro biomarkers)	[256]
Titanium dioxide NPs	TiO₂ NPs (15, 50, 100 nm)	Activates TFEB-dependent autophagy followed by lysosomal dysfunction and ICD-associated oxidative stress	Not specified	Enhanced autophagic response with flux blockade	ICD-associated immunogenic stress	[257]
**Zinc oxide NPs**	ZnO NPs	Triggers zinc ion-ROS-JNK loop causing autophagic cell death and ICD-associated DAMP exposure	PC12 cells (neurotoxicity model)	Neurotoxicity via autophagy-dependent stress pathways	ICD-associated immunogenic stress (in vitro)	[258]
Gold NPs	Au@MC38 (biogenetic gold NPs)	Enhances radiation-induced ROS, autophagy, and ICD with downstream immune activation	Not specified	Tumor growth inhibition; increased CD8α⁺ DCs; synergy with ICB	Functionally validated ICD (in vivo immune activation)	[259]
Iron oxide NPs	ION-loaded macrophages	Promotes ATP/HMGB1 secretion and autophagy-associated stress to enhance radiotherapy-induced immunogenicity	Syngeneic tumor models	Increased DC and cytotoxic T-cell infiltration; enhanced radiotherapy efficacy; improved immune activation	Functionally validated ICD	[260]
Chitosan NPs	ATP/CSO@ECM (chitosan nanocomplexes with engineered cell membrane)	Mimics ICD-associated DAMP cascade; modulates autophagy via proton buffering in acidic TME	Not specified	Prolonged immunotherapy efficacy; enhanced DC activation; robust antitumor immune response	Partial ICD (DAMPs + immune correlates)	[261]
Dendrimers	G4P–C7A (PAMAM derivative with heterocyclic rings)	Induces ER/mitochondrial ROS-driven immunogenic cell death; functions as tumor vaccine	CT26 and 4T1 tumors	Potent tumor growth inhibition; robust antitumor immunity with aPD-1/aCD47 synergy; effective as vaccine	Functionally validated ICD (vaccination-like immunity)	[262]
**Silica NPs**	LDHA@MIP-DSD (biodegradable silica NP with doxorubicin and silybin)	Inhibits LDHA; induces autophagic tumor stress and ICD-associated DAMP release	4T1 breast cancer	Increased CD8^+^ T-cell infiltration; prolonged survival; strong antitumor immune responses	Functionally validated ICD	[263]
Metal NPs	MnOx@MIL-100@CDDP@HA (MMCH)	Induces ferroptosis-mediated ICD; activates cGAS–STING via Mn²⁺; modulates autophagy via ROS	Colorectal cancer	Enhanced antitumor immunity; increased type-I IFN production; significant tumor regression	Functionally validated ICD	[264]
Protein NPs	BSA-Man@Mn²⁺-Ft@Lap	β-lapachone–induced ICD-associated DAMPs; Mn²⁺ enhances cGAS–STING; autophagy via ROS	Multiple murine models (not specified)	Robust DC maturation and T-cell priming; significant tumor regression; non-toxic and tumor-selective	Functionally validated ICD	[265]
Lipid NPs	EM@REV@DOX nanovesicles	DOX-mediated ICD; REV enhances cGAS–STING; autophagy-related immune modulation	Not specified	Enhanced DC maturation, macrophage polarization, and T-cell activation; inhibited distant tumor growth with αPD-L1; prolonged survival	Functionally validated ICD	[266]
Dopamine NPs	PDA NPs (combined with organic/inorganic nanomaterials)	Inhibits pro-survival autophagy during PTT/CT; enhances ICD-associated DAMP release	Not specified	Improved PTT/CT efficacy; enhanced antitumor effects through autophagy inhibition and potential immune activation	Partial ICD (stress + therapy synergy)	[267]
Polymer-lipid hybrid	P/LNVs (cationic polymer-lipid nanovesicles with DOX and siRNA)	DOX-induced PARP1-dependent immunogenic apoptosis; PD-L1 silencing; autophagy via stress	B16 melanoma	Robust CD8 + T-cell responses; 30% tumor eradication in established tumors; effective in prophylactic and metastatic settings	Functionally validated ICD	[268]
Polymer NPs	PEI-PDC (platinum-based polyethylenimine polymer-drug conjugate)	Induces necrotic death with protective autophagy; elicits ICD-associated immune signaling	Not specified	Selective anti-CSC activity; partial reversion of stem-like phenotype; potential for immune-mediated tumor control	Partial ICD	[269]
Polymer NPs	PEG/PEI/CAD NPs (pH-responsive PEI with cis-aconityl-DOX)	DOX-mediated ICD-associated DAMP release; autophagy via pH-triggered release; ICB synergy	Not specified	Enhanced DC recruitment and T-cell activity; excellent therapeutic effect with aPD-1; sustained antitumor immunity	Functionally validated ICD	[270]
Black phosphorus nanosheets	BDM (BP@Decitabine@MDSC membrane vesicles)	PTT/PDT-induced DAMP release; autophagy via mitochondrial damage	Not specified	Significant tumor growth inhibition; reduced MDSC/M2-macrophage infiltration; increased CD4+/CD8 + T-cell and CD103 + DC proportions	Partial ICD (immune remodeling)	[271]
ZIF-NPs	ZIF-8 NPs (SZP: STF62247@ZIF8/PEG-FA; SBZP: STF62247- BMS202@ZIF8/PEG-FA)	Amplifies autophagy toward pro-death; induces ICD-associated immunogenicity	Residual tumors post-IRFA	SZP NPs inhibit proliferation, establish immunological memory; SBZP NPs enhance ICD, activate immune microenvironment, synergize with anti-PD-1/PD-L1	Functionally validated ICD	[272]
ZIF-NPs	mRDZ (Macrophage membrane-camouflaged ZIF-8 with DOX, siIDO1)	Autophagy enhancement via IDO1 silencing; DOX-mediated ICD-associated stress	Solid tumors (lung metastasis)	Suppresses tumor growth, metastasis, and recurrence; induces local/systemic immune responses with immunological memory	Functionally validated ICD	[273]

Note: ICD classification is based on the level of experimental validation reported in the original studies. Detection of isolated DAMPs or in vitro immune correlates is considered indicative of ICD-associated immunogenic stress but does not alone establish functional ICD

## Combination therapy

The complexity of cancer, characterized by tumor heterogeneity, resistance mechanisms, and immunosuppressive TMEs, necessitates multi-pronged therapeutic strategies to achieve robust clinical outcomes [274, 275]. Nanomaterials, with their ability to modulate autophagy and ICD, have emerged as pivotal platforms for combination therapies, integrating modalities such as radiotherapy, PTT, PDT, chemotherapy, and immunotherapy. Utilizing their distinctive physicochemical characteristics, including targeted administration, ROS production, and adjustable functionalization, nanomaterials enhance the synergy of various treatments, thereby strengthening antitumor immunity and overcoming resistance [276, 277]. This section explores recent advances in combination therapies utilizing nanomaterials to modulate autophagy and ICD, highlighting their mechanisms and therapeutic outcomes.

**Fig. 9:**
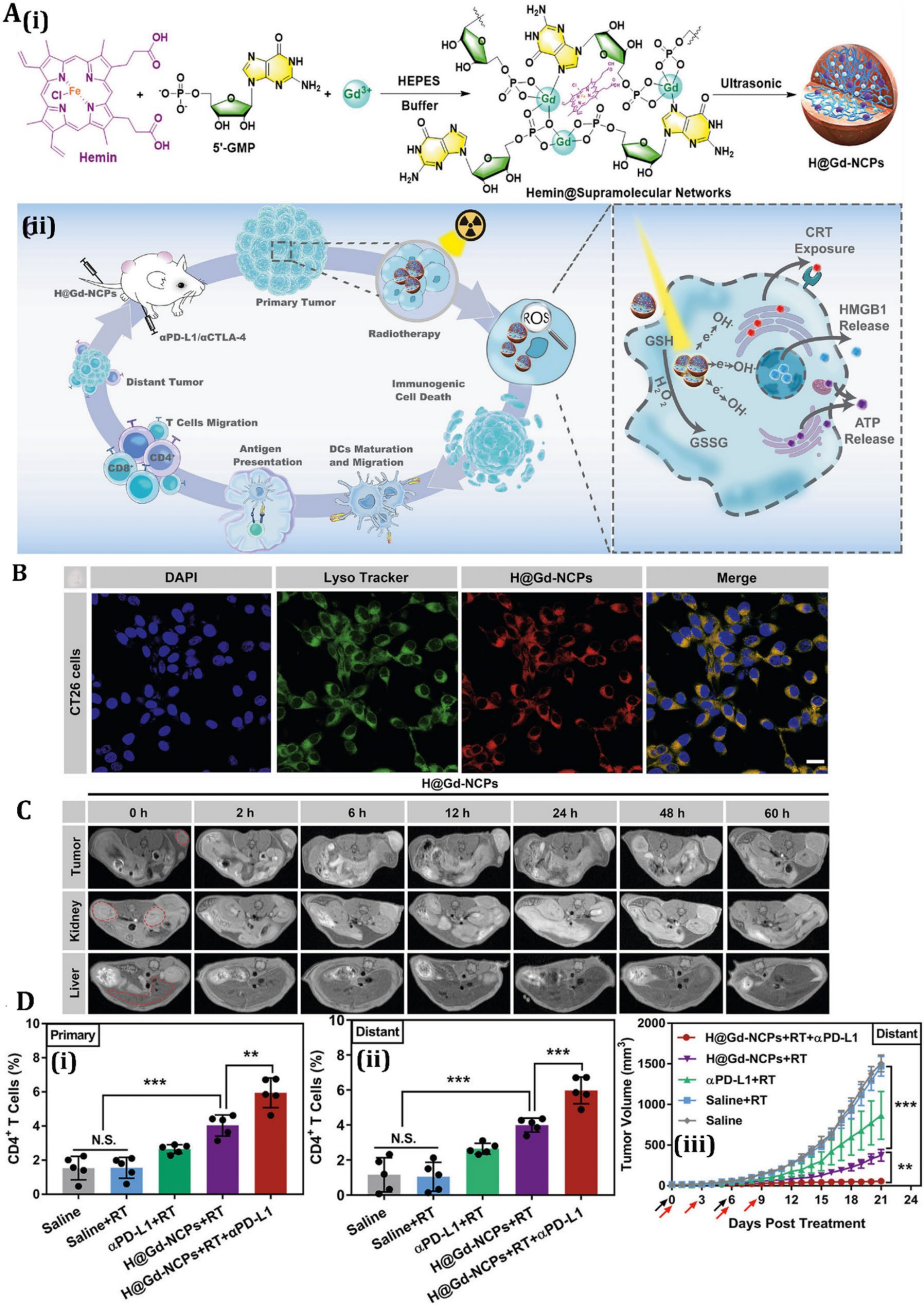
Hemoglobin-loaded gadolinium nanoscale coordination polymers (H@Gd-NCPs) amplify radiotherapy-induced oxidative stress and synergize with immune-checkpoint blockade. **(A)** Schematic overview. Left: one-pot assembly of H@Gd-NCPs. Right: under X-ray irradiation the particles catalyze GSH → GSSG conversion and •OH generation, heightening intracellular oxidative stress and provoking immunogenic cell death (CRT exposure, ATP and HMGB1 release). Resulting dendritic-cell activation potentiates checkpoint inhibitors. (**B**) Cellular uptake and radiosensitization in vitro. Confocal micrographs of CT26 cells stained with DAPI and LysoTracker show H@Gd-NCP fluorescence (yellow overlap) confined to lysosomes, confirming efficient internalization. (**C**) In vivo MR imaging after i.v. injection of H@Gd-NCPs (30 mg Gd kg⁻¹). T1-weighted scans highlight signal enhancement in tumor, kidney, and liver. (**D**) Systemic antitumor immunity and abscopal effect. (i, ii) Flow-cytometry quantification of CD4⁺ T-cell infiltration in irradiated primary tumors (***P = 0.0003, **P = 0.0039) and non-irradiated distant tumors (***P = 0.0003, ***P = 0.0009) after H@Gd-NCP-sensitized RT + checkpoint blockade (*n* = 5). (iii) Growth curves of distant CT26 tumors (**P = 0.002, ***P = 0.0001) demonstrate robust abscopal inhibition with the combination therapy. Reprinted from [284] with permission from Nature.

Radiotherapy uses ionizing radiation, such as X-rays and gamma rays, to interact with cells, causing therapeutic effects through direct or indirect mechanisms. Initially, the efficacy of radiotherapy was attributed to its direct cytotoxic or cytostatic effects on cells [278]. However, recent studies show that radiotherapy’s benefits extend beyond direct cell damage, encompassing local and systemic bystander effects that enhance its immunological impact [279, 280]. Radiation triggers DNA damage, initiating ER stress responses, leading to ICD. This is characterized by the release or surface exposure of DAMPs, which migrate extracellularly, stimulating immune responses [281]. Radiotherapy also promotes the secretion of pro-inflammatory cytokines and chemokines, driving DC maturation and altering the tumor phenotype [282, 283]. Nanomaterials can enhance radiotherapy by sensitizing tumors to radiation-induced damage while promoting ICD and modulating autophagy. For instance, researchers introduced a novel strategy to enhance radiation therapy by using gadolinium-hemin based nanoscale coordination polymers (H@Gd-NCPs) to amplify oxidative stress and induce ICD in tumor cells [284]. These H@Gd-NCPs, synthesized via supramolecular self-assembly, integrate gadolinium for enhanced X-ray absorption and hemin for peroxidase-like activity to deplete glutathione (GSH), thereby increasing ROS and hydroxyl radical (•OH) production. This approach sensitizes radiotherapy, leading to increased ICD markers (e.g., calreticulin exposure, HMGB1 release) and activation of DCs, which prime systemic anti-tumor immune responses (Fig. 9). When combined with checkpoint blockade immunotherapy (e.g., anti-CTLA-4), H@Gd-NCPs significantly inhibit primary, distant, and metastatic tumors in preclinical colorectal and breast cancer models, demonstrating biocompatibility and therapeutic efficacy, with potential for clinical translation in cancer radio-immunotherapy [284]. In another research work, nanoscale metal-organic frameworks (nMOFs) were introduced as effective radiosensitizers for radiotherapy-radiodynamic therapy [285]. A Hf-DBP nMOF was modified to co-deliver imiquimod (IMD) and anti-CD47 antibody (αCD47), allowing macrophage modulation and reversal of immunosuppression in tumors. When synergized with an anti-PD-L1 ICI, this nanoplatform effectively modulates the TME and activates adaptive immunity, resulting in the total elimination of tumors in a bilateral colorectal tumor model [285]. These reinvestigations indicate how nanomaterials, by integrating radiosensitization, autophagy modulation, and ICD induction, transform radiotherapy into a potent immunotherapeutic modality when combined with checkpoint blockade or other immune activators.

**Fig. 10:**
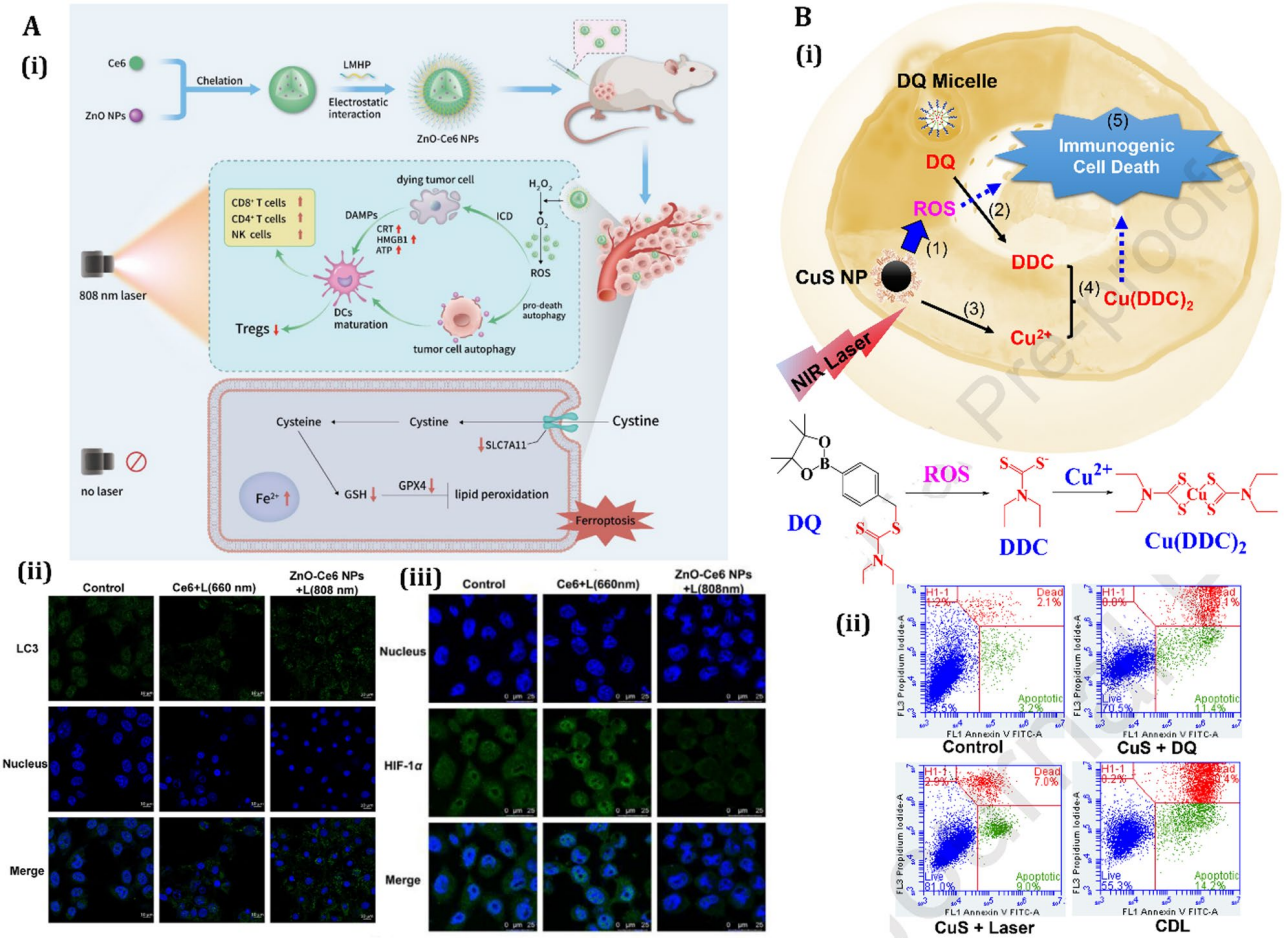
Synergistic induction of photodynamic therapy, ferroptosis, autophagy, and ICD by nanoplatforms activated by near-infrared (NIR) light. **(A)** ZnO-Ce6 nanoplatform-mediated antitumor strategy: **(i)** Schematic representation of ZnO-Ce6 NPs under 808 nm NIR irradiation enhancing PDT by boosting ROS and oxygen availability, triggering pro-death autophagy, and initiating ICD and systemic immune responses. Even without irradiation, ZnO-Ce6 NPs induce ferroptosis via iron overload, downregulation of SLC7A11, GSH, and GPX4, and increased lipid peroxidation. **(ii)** In vitro PDT efficiency: expression of HIF-1α under hypoxic conditions. **(iii)** Confocal imaging of LC3 expression reveals autophagy overactivation in 4T1 cells treated with NIR-irradiated ZnO-Ce6 NPs. Reprinted from [289] with permission from Elsevier. **(B)** NIR-triggered prodrug-based chemoimmunotherapy: **(i)** Mechanism of CDL nanoplatform action: NIR-induced ROS (via CuS NPs) activates DQ prodrug into DDC; simultaneously released Cu²⁺ forms cytotoxic Cu(DDC)₂, achieving dual-mode chemotherapy and ROS-driven ICD. **(ii)** ROS levels measured in 4T1 cells via DCFH-DA assay under various treatments. Apoptosis was determined via Annexin V/PI staining, distinguishing early (Annexin V⁺/PI⁻) and late-stage (Annexin V⁺/PI⁺) apoptotic cells. Reprinted from [291] with permission from Elsevier

PTT and PDT, which harness light-activated nanomaterials, are increasingly integrated with chemotherapy, immunotherapy, or autophagy modulators to enhance ICD and regulate autophagy, offering synergistic anticancer effects [286]. PDT relies on photosensitizers (PS) that generate ROS, including singlet oxygen (¹O₂) and hydroxyl radicals (·OH), upon light activation, inducing oxidative stress and cell death [32]. Tumor cells, with their low oxidative stress tolerance, are highly susceptible to this ROS-mediated damage. Hypericin-based PDT, a potent Type II ICD inducer, triggers ER stress by disrupting ATP2A2-mediated calcium homeostasis, leading to CRT exposure and ICD [287]. Photosensitizers like phenothiazine dyes, cyanines (e.g., merocyanine 540), and polycyclic compounds (e.g., hypericin, hypocrellin) selectively damage organelles such as the ER, mitochondria, or lysosomes, releasing tumor debris that amplifies ICD through DAMP release [288]. A notable example involves the development of biocompatible zinc oxide nanoparticles (ZnO NPs) integrated with chlorin e6 (Ce6) to form ZnO-Ce6 NPs, which enhance PDT by addressing its limitations of poor tissue penetration and tumor hypoxia [289]. These nanoparticles exhibit upconversion properties, converting 808 nm near-infrared light to 401 nm visible light to excite Ce6, enabling deeper tissue penetration, and possess catalase-like nanozyme activity to decompose hydrogen peroxide into oxygen, alleviating hypoxia (Fig. 10**A**). The enhanced PDT generates ROS, over-activating autophagy, inducing ICD, and triggering ferroptosis even without light, leading to significant antitumor effects in 4T1 breast cancer models [289]. In vivo results showed substantial tumor growth inhibition, with some tumors completely eradicated, highlighting ZnO-Ce6 NPs’ potential as a multifunctional platform for cancer therapy by combining PDT, autophagy, ferroptosis, and antitumor immunity. Another interesting work presented a novel approach to targeting tumor cells and ER using a novel photosensitized ICD inducer, CET, and autophagy inhibitor chloroquine (CQ) [290]. The material, based on poly-β-cyclodextrin, has excellent solubility and stability. The ICD effect is enhanced by CET’s enrichment in the ER and autophagy inhibition caused by CQ. The study shows that Da-CD@CET@CQ NPs effectively inhibit tumor growth and metastasis, providing a valuable guideline for targeted-PDT-synergistic immunotherapy of TNBC [290]. This innovative approach offers a promising strategy for targeted-PDT-synergistic immunotherapy. Another study developed a novel combination therapy using copper sulfide nanoparticles, disulfiram prodrug (DQ), and NIR laser to improve tumor specificity and enhance anticancer efficacy [291]. The CuS NPs combined with NIR laser therapy selectively activate the DQ micelle at the tumor site, resulting in the release of DDC and the in situ formation of Cu (DDC)_2_. The therapy uses various mechanisms, such as Cu (DDC)_2_ chemotherapy and the ROS amplification cascade, to kill tumor cells. The NIR light-triggered tumor-specific transition reduces systemic toxicity (Fig. 10B) [291].

Sonodynamic therapy (SDT) has emerged as a potential approach in cancer treatment, utilizing ultrasound-activated sonosensitizers to produce ROS that elicit localized cytotoxicity and initiate ICD [292]. In contrast to PDT, which is controlled by superficial tissue penetration, SDT employs ultrasound’s greater depth of penetration to activate sonosensitizers, generating ROS via a cavitation effect characterized by the formation, expansion, and collapse of bubbles under extreme conditions (temperatures exceeding 5000 K and pressures surpassing 800 atm) [293, 294]. This mechanism activates sonosensitizers, including organic chemicals (e.g., porphyrin derivatives, Rose Bengal, doxorubicin, and erythrosine) and inorganic materials, to cause ICD and promote antitumor immunity by ROS-mediated apoptosis [295]. While PDT is currently supported by more mature preclinical and early clinical evidence for ICD induction, particularly in terms of robust DAMP release and synergy with immune checkpoint blockade, its therapeutic performance remains highly dependent on light accessibility and oxygen availability. In comparison, SDT offers a distinct advantage for inducing ICD in deeply located tumors; however, most SDT-based ICD studies remain at the preclinical stage and require further in vivo and translational validation. The selection of sonosensitizer is crucial, as organic alternatives are frequently constrained by brief circulation durations and inadequate tumor retention owing to their lipophilic characteristics [296].

Xue et al. devised an SDT method that targets mitochondria with IR780, utilizing ultrasound to augment ROS production, which enhances CRT exposure and HMGB1 release, hence amplifying ICD and immunological responses [243]. Nanoparticles have distinct physicochemical features that can be utilized to develop extremely efficient acoustic sensitizers. For instance, a novel nanoplatform, ZrO2-x@PEG/cRGD was developed for the first time as a therapy-activated ICD inducer to enhance photothermal-augmented sonodynamic tumor eradication within the NIR-II biological window [297]. A photothermal conversion efficiency of 45.8% was achieved for photothermal therapy using the ZPR nanoparticles, which exhibited strong optical absorbance. ROS generated during sonodynamic therapy can elicit ICD, enhancing systemic anti-tumor immunity and resulting in full tumor eradication after treatment [297]. Ultrasmall iron-doped titanium oxide nanodots (Fe-TiO_2_ NDs) have also been investigated to enhance SDT and offer Fenton-catalytic function for chemodynamic therapy [298]. PEGylated CoFe_2_O_4_ nanoflowers (CFP) have also been developed as bioreactors for augmented SDT and chemodynamic therapy to elicit robust immune responses [299]. CFP exhibits strong Fenton-like and catalase-like activity and can generate ROS for SDT and chemodynamic therapy [299]. This approach can trigger ICD, suppressing primary and distant tumors and lung metastasis. Overall, based on current preclinical evidence, the combinations that most consistently induce robust ICD (multiple DAMPs release, DC/T-cell activation, abscopal effects, metastasis control, or ICB synergy) involve: (i) PDT with immunotherapy using ER-targeting or autophagy-modulating nanoplatforms; (ii) radiotherapy with checkpoint blockade enhanced by radiosensitizing nanomaterials; and (iii) SDT combined with PTT or immunotherapy using mitochondria-targeted or multifunctional nanoplatforms. These findings provide a preclinical framework for selecting optimal combination strategies to maximize ICD, although clinical validation remains necessary.

It should be also noted that several technical and biological barriers must still be overcome to achieve successful clinical translation. These include limited clinical experience with SDT protocols, challenges in precisely controlling ROS dosage and spatial distribution, insufficient tumor retention and circulation time of sonosensitizers, and the highly context-dependent role of autophagy, particularly in heterogeneous tumors and cancer stem cell populations [296, 300]. Additionally, the lack of standardized ICD biomarkers and well-validated clinical endpoints continues to hinder reliable translational evaluation. Addressing these challenges will be critical to unlocking the full therapeutic promise of these emerging nanoplatforms.

The overview of recent studies that investigated different combination therapies for modulating autophagy and ICD is summarized in Table 2.

**Table 2:** Combination Therapies Utilizing Nanomaterials for Autophagy and ICD Modulation in Cancer Treatment

Nanomaterial type	Formulation	Mechanism of action	Cancer model	Key outcomes	Ref.
Polymer NPs	TPAQ-Py-PF6 + paclitaxel nanoplatform	Combines PDT and chemotherapy; induces ICD via cGAS-STING activation (cytokine release); triggers autophagy via ROS; enhances immunotherapy	Not specified	Enhanced tumor targeting; systemic immune response; DC maturation and T-cell recruitment	[301]
Gold NPs	Au-DOX@PO-ANG polymersomes	Combines chemotherapy and radiotherapy; induces ICD (CRT, HMGB1, ATP) and autophagy via DOX and radiation stress; promotes DC maturation	U87-MG, G422 (glioma)	Efficient vaccination potential; enhanced DC maturation; synergistic chemoradiotherapy effects	[302]
Gadolinium NPs	AGuIX NPs (< 5 nm)	Enhances radiotherapy via radiosensitization; induces ICD and autophagy via radiation-induced DNA damage and ROS; synergizes with anti-PD-1	B16 melanoma	Increased CD8 + T-cell infiltration; alleviated immunosuppressive TME; durable antitumor T-cell responses	[303]
Copper sulfide NPs	CuS nanosheets	Combines SDT and catalytic therapy (carbon monoxide (CO), ROS); induces ICD via ROS and CO-mediated mitochondrial damage; triggers autophagy via ROS; reverses hypoxia and immunosuppression	Not specified	Regressed primary/distal tumors; inhibited lung metastasis; enhanced T-cell infiltration and DC maturation	[304]
Polydopamine NPs	CCP@HP@M (Ce6, chloroquine, Pt nanozymes in hollow polydopamine)	Combines SDT and autophagy inhibition; induces ferroptosis; inhibits protective autophagy with chloroquine; enhances apoptosis	Colorectal cancer	Enhanced tumor cell elimination; alleviated hypoxia; stimulated apoptosis and ferroptosis; suppressed tumor growth	[305]
Mesoporous organosilica	HHBP (HMME@HMONs-3BP-PEG)	Combines SDT and respiration inhibition; induces ROS-mediated apoptosis; overactivates pro-death autophagy via 3BP; alleviates hypoxia	Not specified	Remarkable antitumor activity; alleviated tumor hypoxia; excessive autophagy enhanced efficacy; reduced metastasis	[306]
Metal-organic framework	Cur@MOF-GOx/HA (GOx, curcumin on NH2-MIL88)	Combines starvation and chemodynamic therapy; glucose depletion; overactivates pro-death autophagy with curcumin; enhances cell death	Not specified	Enhanced starvation therapy; restrained metastasis; synergistic starvation, autophagy, and chemodynamic effects	[307]
N-TiO2 Nanoparticles	Nitrogen-doped TiO2 in PDT	Photo-activated ROS production; induces autophagy flux (low dose) or autophagosome-lysosome fusion impairment (high dose)	Melanoma (A375)	Nontoxic autophagy flux in dark; photo-activation triggers ROS and autophagy blockade, enhancing ICD	[308]
N-TiO2 Nanoparticles	Nitrogen-doped TiO2 in PDT	ROS-dependent autophagy induction; triggers differentiation or apoptosis based on ROS levels	Leukemia (K562)	Autophagy-dependent differentiation at low ROS; apoptosis at high ROS, potential for ICD	[309]

## Clinical translation and future directions

Nanomaterial-based approaches utilizing the interplay between autophagy and ICD provide a novel strategy for cancer immunotherapy. Nanomaterials offer compelling approaches to overcome immune evasion and resistance in solid tumors by enabling precise regulation of tumor immunogenicity. Despite preclinical studies demonstrating their ability to enhance anti-tumor immunity via the targeted delivery of autophagy modulators and inducers of ICD, several substantial challenges hinder their practical use [310–312].

Biosafety issues related to certain categories of nanomaterials, particularly metallic nanoparticles like AgNPs and IONPs, pose a significant obstacle [313]. The release of cytotoxic silver ions from AgNPs may induce systemic oxidative stress and off-target organ accumulation, while IONP-induced ROS, although essential for ICD activation, can disrupt mitochondrial function and damage DNA [314, 315]. The TME, defined by hypoxia, an acidic pH (5.5–6.5), and immunosuppressive cytokines like TGF-β, can impede DAMP signaling and limit immune cell infiltration, consequently reducing the effectiveness of ICD-based therapies [316, 317]. Tumor heterogeneity presents challenges: cancer stem cells often rely on autophagy for survival, but differentiated tumor cells may have suppressed autophagy due to mTORC1 hyperactivation [318–320]. Such disparities necessitate personalized, context-specific nanomaterial design.

Importantly, although ICD attempts to provoke a strong antitumor immune response, prolonged or excessive release of DAMPs may, unexpectedly, diminish therapeutic effectiveness. Prolonged extracellular exposure to DAMPs like ATP and HMGB1 can induce sustained inflammation, attract immunosuppressive myeloid cells, and activate compensatory immune-regulatory mechanisms within the tumor microenvironment [321]. Prolonged ATP signaling may result in P2RX7 desensitization or enhanced conversion to immunosuppressive adenosine through CD39/CD73 activity, ultimately promoting immunological tolerance. Similarly, prolonged HMGB1-TLR4 signaling has been linked to T cell malfunction and exhaustion in chronic inflammatory states [322–324]. These observations indicate the necessity for dynamic regulation of ICD induction to enhance immunogenicity while preventing chronic inflammation and immunological exhaustion, a balance essential for practical application.

Translational progress is also impeded by regulatory and manufacturing barriers. Stimuli-responsive nanomaterials (e.g. pH- or ROS-sensitive polymers) remain harder to reproduce and scale than established products such as liposomal doxorubicin [325, 326]. While the lack of standardized ICD and autophagy biomarkers presents a barrier to clinical translation, emerging strategies may enable patient-specific therapies. Multiparametric profiling of ICD (CRT exposure, HMGB1, ATP release) and autophagy (LC3-II, p62), together with immunological parameters such as cytokine levels and immune cell infiltration, could help identify patients most likely to respond to specific combinations [327, 328]. Nanoplatforms incorporating imaging or biosensing capabilities, integrated with AI-driven modeling, offer real-time monitoring of autophagy modulation and ICD induction, allowing functional, rather than purely molecular, stratification. Additionally, adaptive trial designs using these dynamic readouts could optimize combination therapies for each patient, while biomimetic or stimuli-responsive nanomaterials (e.g., tumor membrane-coated or ROS/pH-sensitive carriers) ensure targeted and context-specific ICD induction.

Notwithstanding the promise demonstrated in first trials utilizing combinations of pegylated liposomal doxorubicin and ICIs, challenges remain concerning dosage optimization, treatment preparation, and patient-specific variability [223, 329]. To address these translational limitations, innovative design strategies are integrating bioinspired characteristics with advanced materials engineering to enhance safety, specificity, and regulatory viability. Surface PEGylation, frequently utilized to diminish immunogenicity and extend circulation duration, can mitigate the cytotoxicity of nanomaterials by minimizing oxidative stress and improving systemic tolerance [330].

In addition to PEG, tumor-derived membrane coatings represent an effective approach for enhancing tumor selectivity and evading immune clearance [331–333]. An example of this phenomenon is the selective accumulation of chitosan-coated AgNPs decorated with breast cancer cell membranes in TNBC models. These nanoparticles effectively induce necroptosis-driven ICD with minimal systemic toxicity [334]. These biomimetic coatings specifically target tumors by the utilization of homotypic adhesion molecules. Similarly, exosome-mimetic nanovesicles originating from tumor cells exhibit potential in co-delivering autophagy inhibitors, such as chloroquine, to acidic TME, hence augmenting DAMP exposure and immune activation [335–337].

Zwitterionic materials, such as phosphorylcholine-based polymers, reduce protein corona formation and extend their retention in systemic circulation to address biodistribution challenges [338, 339]. Biodegradable nanoparticles incorporating disulfide linkers or ester bonds undergo biological degradation in the TME into inert byproducts under reductive or enzymatic conditions, hence offering an additional safety layer [340, 341]. For instance, disulfide-bridged polymeric carriers loaded with ICD inducers, such as SHK, deliver their payload exclusively to tumor sites to prevent accumulation in healthy tissues [342].

TME-responsive systems, such as pH-sensitive, redox-responsive, and enzyme-triggered platforms, provide accuracy in regulating autophagy and ICD [343]. These systems utilize acidic pH or elevated ROS levels in the TME to trigger on-demand release of payloads, improving therapeutic efficacy while minimizing off-target effects. The integration of artificial intelligence (AI)-driven modeling with theranostic agents (e.g., MRI-visible iron oxide nanoplatforms) as well as non-invasive biomarkers enables real-time monitoring, patient stratification, and personalized treatment [344, 345].

Collectively, these examples outline a design-oriented framework in which nanomaterial composition (e.g., metallic vs. biodegradable polymers), surface functionalization (e.g., PEGylation, biomimetic coatings), and stimuli-responsiveness (e.g., pH-, redox-, or enzyme-triggered release) jointly determine whether autophagy is exploited to enhance ICD or diverted toward cytoprotective and immune-evasive pathways. Ultimately, these multifunctional nanoplatforms could potentially provide safe and precise treatments for patients with resistant cancers, as long as further research and strict clinical testing are done.

## Supplementary Information

Below is the link to the electronic supplementary material.

Supplementary Material 1

## Data Availability

No datasets were generated or analysed during the current study.
