# Supplementary material for: The interplay between autophagy and immunogenic cell death: nanomaterial-based strategies for cancer immunotherapy
Source: J Nanobiotechnology. 2026 Apr 12;24:490. doi: 10.1186/s12951-026-04286-5 (PMC13200464; doi:10.1186/s12951-026-04286-5)
Supplement: Supplementary file 1 — Supplementary Material 1 [file 12951_2026_4286_MOESM1_ESM.docx]

**Uncropped Gels and Blots image(s)**

**Not applicable**
